# Integrating Genetic and Network Analysis to Characterize Genes Related to Mouse Weight

**DOI:** 10.1371/journal.pgen.0020130

**Published:** 2006-08-18

**Authors:** Anatole Ghazalpour, Sudheer Doss, Bin Zhang, Susanna Wang, Christopher Plaisier, Ruth Castellanos, Alec Brozell, Eric E Schadt, Thomas A Drake, Aldons J Lusis, Steve Horvath

**Affiliations:** 1 Department of Microbiology, Immunology, and Molecular Genetics, University of California Los Angeles, Los Angeles, California, United States of America; 2 Department of Human Genetics, David Geffen School of Medicine, University of California Los Angeles, Los Angeles, California, United States of America; 3 Department of Biostatistics, School of Public Health, University of California Los Angeles, Los Angeles, California, United States of America; 4 Rosetta Inpharmatics, Inc., Kirkland, Washington, United States of America; 5 Department of Pathology and Laboratory Medicine, David Geffen School of Medicine, University of California Los Angeles, Los Angeles, California, United States of America; 6 Department of Medicine, David Geffen School of Medicine, University of California Los Angeles, Los Angeles, California, United States of America; 7 Molecular Biology Institute, University of California Los Angeles, Los Angeles, California, United States of America; North Carolina State University, United States of America

## Abstract

Systems biology approaches that are based on the genetics of gene expression have been fruitful in identifying genetic regulatory loci related to complex traits. We use microarray and genetic marker data from an F2 mouse intercross to examine the large-scale organization of the gene co-expression network in liver, and annotate several gene modules in terms of 22 physiological traits. We identify chromosomal loci (referred to as module quantitative trait loci, mQTL) that perturb the modules and describe a novel approach that integrates network properties with genetic marker information to model gene/trait relationships. Specifically, using the mQTL and the intramodular connectivity of a body weight–related module, we describe which factors determine the relationship between gene expression profiles and weight. Our approach results in the identification of genetic targets that influence gene modules (pathways) that are related to the clinical phenotypes of interest.

## Synopsis

Obesity is a major pub lic health concern in many developed countries. While some people appear to stay lean no matter what or how much they eat, others appear to be genetically predisposed to obesity. The genetic similarity between mouse and human makes the mouse a promising mammalian model system to study obesity. Advantages of mouse models include the ability to control diet/environment and easy access to relevant tissues for gene expression studies. Mouse cross studies have implicated dozens of chromosomal regions that contain weight-predisposing genes, and gene expression studies have yielded hundreds of body weight–related genes. In this study, the authors use a gene network–based approach for integrating clinical traits, genetic marker data, and gene expression data. Instead of focusing on individual genes, the authors provide a systems-level view of a module of genes related to body weight. The resulting model allows them to characterize weight-related genes utilizing network concepts (intramodular connectivity) and genetic concepts (module quantitative trait locus). This integrative genomics approach provides new insights into the relationship between gene expression and body weight.

## Introduction

The identification of pathways and genes underlying complex traits using standard mapping techniques has been difficult due to genetic heterogeneity, epistatic interactions, and environmental factors [1,2]. One promising approach to this problem involves the integration of genetics and gene expression [3–7]. Network methods have been applied to identify and characterize various biological interactions [8–11], and have helped to predict gene function in lower eukaryotes [12,13]. Recently, gene network methods have been used in the analysis of complex traits in higher organisms [14,15]. Here, we present a novel approach for using gene co-expression networks to study the genetics of complex physiological traits that are relevant to metabolic syndrome (obesity, insulin resistance, and dyslipidemia).

The traditional quantitative trait locus (QTL) mapping approach [16] relates clinical traits to genetic markers directly. Several authors have proposed a strategy that uses genome-wide gene expression data to help map clinical traits [3–7,17–25] The strategy uses the genetics of gene expression to reconstruct metabolic or regulatory pathways, and utilizes gene expression as a quantitative trait, thereby mapping expression QTL (eQTL). Using this method, several groups have found “hotspot” regions (regulatory gene regions) in the genome that are involved in regulating the expression of many genes [3,18,26]. These hotspots highlight the genomic loci that determine the relationship between the phenotype and groups of functionally related genes [26]. Here, we propose a novel approach for integrating network properties with genetic information to determine the relationship between clinical traits and groups of physiologically relevant genes. The following steps summarize our overall approach: (1) A gene co-expression network is constructed from genome-wide expression data from a segregating population. (2) The biochemical and physiological significance of the network modules are determined. (3) Genetic loci regulating gene modules within the network are identified. (4) Network properties are integrated with genetic information to explain the biological significance of the module genes.

## Results

### Construction of a Weighted Mouse Liver Co-Expression Network

The details of the gene co-expression network construction are given in [27] and summarized in Materials and Methods. Briefly, the absolute value of the Pearson correlation coefficient is calculated for all pair-wise comparisons of gene expression values across all microarray samples. This correlation matrix is then transformed into a matrix of connection strengths using a power function (connection strength = |correlation|^ß^), resulting in a weighted network. The use of weighted networks represents an improvement over unweighted networks that are based on dichotomizing the correlation matrix, because (1) the continuous nature of the gene co-expression information is preserved and (2) the results of weighted network analyses are highly robust with respect to the choice of the parameter ß, whereas unweighted networks display sensitivity to the choice of the cutoff threshold [27].

We applied the network construction algorithm to a subset of gene expression data from an F2 intercross between inbred strains *C3H/HeJ* and *C57BL/6J.* Liver gene expression data from 135 female mice were used for this analysis (see Materials and Methods). This cross is described in detail elsewhere [28], and will herein be referred to as the BxH cross.

**Figure 1:**
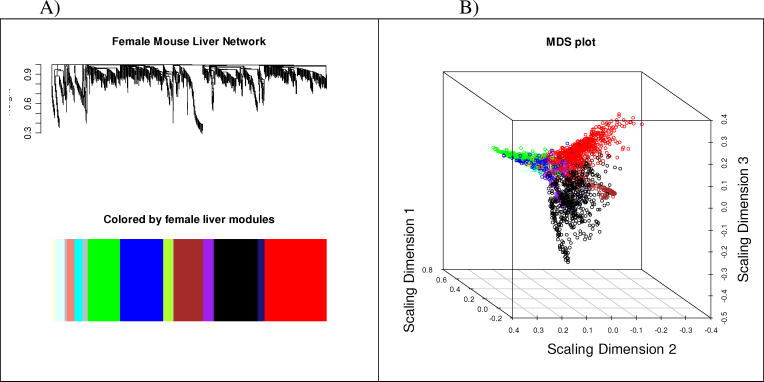
Visual Representations of the Gene Co-Expression Network (A) Hierarchical clustering of 3,421 genes and visualization of gene module partitioning. The colored bars (below) directly correspond to the module (color) designation for the clusters of genes. One can visualize where in the whole clustering dendrogram the gene modules were defined. (B) Multi-dimensional scaling plot representation of the entire gene expression network. Each gene is represented by a dot, where the color of the dot corresponds to the gene module to which that gene belongs. The distance between each dot is representative of their topological overlap. This representation allows one to visualize the relationships among genes within a gene module, how that module is related to the rest of the network, and how closely any two modules are related.

As detailed in the Materials and Methods section, we used only the 3,421 most varying and most connected genes for module detection. This should not lead to major information loss since module genes tend to be highly connected. As shown in Figure 1A and 1B, transcripts were clustered into distinct groups, herein referred to as modules. We were able to identify 12 distinct gene modules or groups of genes with high topological overlap (for details on module construction, see Materials and Methods). To distinguish between modules, we designated each module by an arbitrary color. The number of genes included in the modules ranged from 34 (Light-yellow) to 772 (Red), and their mean overall connectivity (k_all_) ranged from 6.49 (Salmon) to 27.58 (Brown). We also defined the intramodular connectivity (k_in_) for each gene based on its Pearson correlation with all other genes in the module (see Materials and Methods). Summary statistics for all gene modules are available in Table S1. Detailed information about all genes and their network properties are available in Protocol S1.

Since we make use of the intramodular connectivity in the Blue module (534 genes), we studied its robustness with respect to dropping half of the microarrays used in its definition. Specifically, the following procedure was carried out 1,000 times. We randomly omitted half of the microarrays from the analysis when computing intramodular connectivity of the Blue module genes. Next, we correlated the resulting connectivity measures with the original full data intramodular connectivities. The median correlation between the full data connectivity and that of the 1,000 replicate subsets was 0.89 (interquartile range = [0.86,0.91]), which demonstrates that the intramodular connectivity measure is highly robust with respect to randomly omitting half of the microarrays.

### Biological Significance of Network Modules

We examined the functional significance of the gene modules by testing for both enriched biochemical pathways and subcellular compartmentalization of proteins corresponding to genes within a gene module. First, we examined whether these modules were enriched for gene products in a specific subcellular compartment as defined by Gene Ontology (GO). Using the WebGestalt web site (http://genereg.ornl.gov/webgestalt) [29], we calculated the enrichment of any specific GO cellular component in each module. All statistically significant observations are presented in Protocol S2. The results show that each module was enriched with distinct gene sets belonging to separate compartments within the cell. Interestingly, very few modules shared the same category. The “nucleus” category was enriched for both the Black and the Green-yellow (light green in color) modules. However, the nuclear genes for the Green-yellow module were genes involved in chromosomal maintenance, and for the Black module, they were genes involved in the regulation of transcription and DNA binding. Overall, these results are consistent with a model in which some gene modules correspond roughly to the spatial location of their products.

In order to gain further insight into the functional significance of the gene modules, we utilized the Expression Analysis Systemic Explorer (EASE) software package [29] which can be used to conduct a pathway enrichment analysis for each module. EASE can be utilized to find enrichment of pathways annotated in the Kyoto Encyclopedia of Genes and Genomes (KEGG). The EASE analysis for functional enrichment of modules found that seven modules are significantly enriched. The Brown module is enriched for genes within the Biosynthesis of steroids (*p* = 2.3 × 10^−6^) and Glycolysis/Gluconeogenesis” (*p* = 1.5 × 10^−5^) pathways. These results are consistent with the finding that the Brown module is closely related to the plasma insulin levels (see below). The Blue module is enriched for genes in the ECM-receptor interaction (*p* = 2.3 × 10^−9^) and Complement and coagulation cascades (*p* = 1.0 × 10^−6^) pathways. The Green module is enriched for the Toll-like receptor signaling (*p* = 1.6 × 10^−8^), the Cytokine–cytokine receptor interaction (*p* = 2.9 × 10^−7^), and the Hematopoietic cell lineage (*p* = 6.6 × 10^−5^) pathways. The Green-yellow module is enriched for genes involved in the Cell cycle pathway (*p* = 6.3 × 10^−18^). The Light-yellow module is enriched for genes involved in the MAPK signaling pathway (*p* = 5.4 × 10^−3^). The Midnight-blue module is enriched for genes involved in the Nitrobenzene degradation pathway (*p* = 7.1 × 10^−4^). The Light-yellow module is enriched for genes involved in the Ribosome pathway (*p* = 3.1 × 10^−4^). A detailed KEGG pathway analysis for each module can be found in Protocol S2.

**Figure 2:**
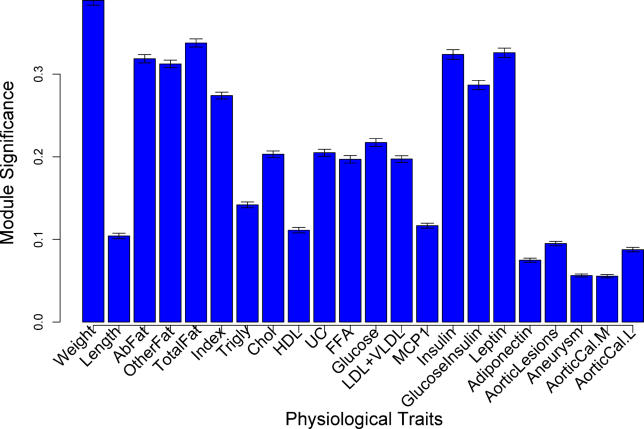
The Relationship between the Blue Module and Several Clinical Traits Module significance is defined as the mean of the absolute value of the correlation coefficient for all genes within a module with a physiological trait of interest. A module significance of 0.30 is statistically significant at a *p*-value of 0.05 for any module given *n* = 135 mice after correction for multiple comparisons (see Materials and Methods). Abfat, abdominal fat (g); Aneurysm, aneurysms as measured by histological examination using a semi-quantitative scoring method. AorticCal.L, aortic calcification in the lesion area; AorticCal.M, medial aortic calcification; Chol, total plasma cholesterol (mg/dl); FFA, plasma free fatty acids (mg/dl); HDL, high-density lipoprotein fraction of cholesterol (mg/dl); Index, adiposity index (total fat × 100/weight); Ins, plasma insulin (μg/l); LDL+VLDL, plasma low-density lipoprotein and very low-density lipoprotein choleseterol (mg/dl); Lesion, aortic lesion size as measured by histological examination using a semi-quantitative scoring method; MCP-1, plasma MCP-1 protein levels; otherfat, subcutaneous, retroperitoneal, and mesenteric fat (g); TG, plasma triglycerides (mg/dl); Totalfat, Abfat + otherfat; UC, plasma unesterified cholesterol (mg/dl).

Next, we assessed the physiological relevance of each module by examining the overall correlation of the module genes with each of the phenotypes in F2 mice. In the BxH F2 cross, 22 physiological traits were measured for each animal. For a given physiological trait, we defined a measure of gene significance by forming the absolute value of the correlation between trait and gene expression values. For example, the body weight can be used to define a gene significance of the i^th^ gene expression GSweight(i) = |cor(x(i), weight)| where x(i) is the gene expression profile of the i^th^ gene. The mean gene significance for a particular module can be considered as a measure of module significance (MS) (see Materials and Methods for statistical test), which means that MS provides a measure for overall correlation between the trait and the module. For example, a module with a high MS value for “body weight” is on average composed of genes highly correlated with body weight. The two highest MS scores observed were for the Blue module with mouse weight (g) (MS = 0.395, *p* = 7.7 × 10^−5^), and the Brown module with plasma glucose-insulin ratio (MS = 0.336, *p* = 0.004). In addition, the Blue module was also related to abdominal fat pad mass (g) (MS = 0.323, *p* = 0.009), and total mass (g) of other fat depots (MS = 0.309, *p* = 0.02; Figure 2). The Brown module was also found to be significantly related to plasma insulin levels (mg/dl) (MS = 0.295, *p* = 0.04). The results for the trait analyses for all modules with all traits are available in Figure S1. Since the Blue module was the most significantly related module to our clinical traits, our follow-up analysis focuses on this module.

It has been demonstrated in lower organisms that targeted attacks on genes with very high connectivity often result in lethal phenotypes [30–33]. These results motivated us to study genes with high values of the intramodular connectivity measure (k_in_). As described in the previous section, the k_in_ for each gene is based on its Pearson correlation with all the other genes in the module (Materials and Methods). To assess the significance of k_in_, we analyzed the Blue module, which showed the highest MS value in our previous analysis, and plotted k_in_ against gene significance for all 534 genes in this module. The plot is shown in Figure S2A (first panel). It is evident from this plot that there is a direct relationship between the connectivity of a gene and the extent to which it is related to the body weight (Spearman correlation = 0.41, *p* < 10^−20^). This analysis showed that the connectivity within a physiologically relevant module is directly related to the gene significance. General information for all Blue module hub genes is presented in Table S2. We also measured gene expression levels and calculated the intramodular connectivity and gene significance for the Blue module genes in the brain, muscle, and adipose tissues in both male and female *(C57BL/6JxC3H/HeJ)* F2 mice. We find a moderate relationship between the connectivity and gene significance in male liver gene expression network (Spearman correlation 0.16 and *p* = 0.00028; Figure S2). In addition, our data suggest negligible relationships between connectivity and gene significance for other tissues (Figure S2). Since our data were not designed for detecting subgroup differences, the tissue-specific conclusion about relationship between connectivity and gene significance warrants further validation.

### Genetic Analysis of the Network Modules

**Figure 3:**
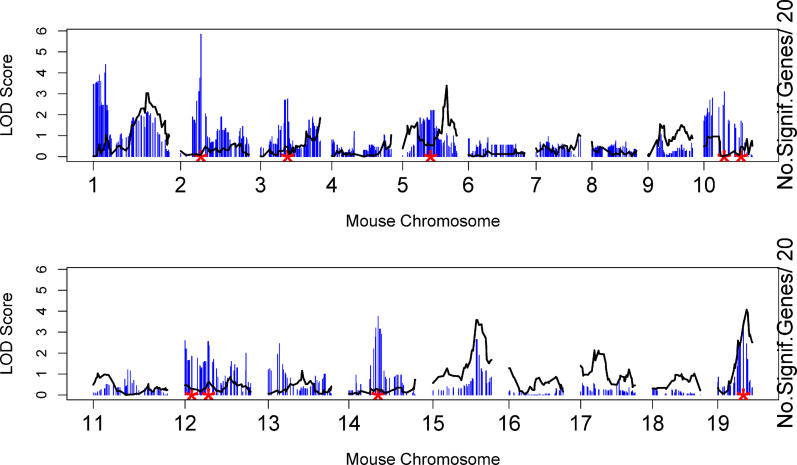
Blue Module mQTL Profile Partially Overlaps with the Physiological Trait (Weight) QTL The black curves represent the QTL analysis across the genome for mouse body weight using a single marker genome scan according to the LOD score scale to the right. Evidence of linkage for weight was found on Chromosome 1 (50.4 Mb, LOD = 3.03), Chromosome 5 (118.5 Mb, LOD = 3.39), Chromosome 15 (78.2 Mb, LOD = 3.58), and Chromosome 19 (50.4 Mb, LOD = 4.07). For each marker, the blue vertical line represents the number (divided by 20) of significant Blue module genes, i.e., the number of genes whose single-point LOD at this marker is bigger than 2. For example, there is a SNP marker on Chromosome 2 at which 117 Blue module genes have a LOD bigger than 2. The red stars denote the locations of the mQTLs reported in Table 1.

The above results regarding co-expression modules made use only of the gene expression data. In the following, we relate the module gene expressions to the genetic marker data to investigate the genetic underpinnings of the network modules. Recently, we and others have shown that there are genomic “hotspots” across the genome that regulate transcript levels of the genes [3,14,18,26]. Since the co-expression network constructed here contained biologically relevant gene modules, we hypothesized that there might also be genomic hot spots that coordinately regulate the transcript levels of the genes within each module. Using 1,065 single nucleotide polymorphism (SNP) markers that were evenly spaced across the genome (~1.5 cM density), we mapped the gene expression values and plotted the distribution of the eQTL [3,7] for all genes within each gene module. We defined a module quantitative trait locus (mQTL) as the locus with a significant enrichment for eQTL of the genes within a predetermined gene module. Since we were interested in studying the genetics of modules as opposed to the genetics of the entire network, when searching for mQTLs, we focused on module-specific eQTL hot spots[3,4]. Toward this end, we used the Fisher exact test to determine whether the proportion of module genes that map to the eQTL hotspot was significantly higher than that of the 8,000 network genes. The results for the Blue module are shown in Figure 3. We found that the Blue module has nine mQTLs. These are located on Chromosomes 2, 3, 5, 10 (middle), 10 (distal), 12 (proximal), 12 (middle), 14, and 19. The physical locations of the peak markers for all mQTL of the Blue module are given in Table 1.

**Table 1:**
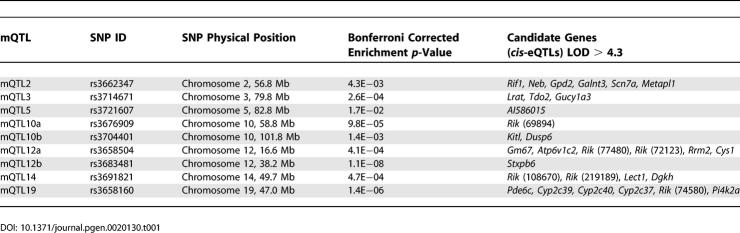
Blue Module mQTL and Candidate *cis*-eQTL at These Loci

To identify the likely candidate genes for each of the mQTL loci, we examined the *cis-*eQTLs at each of these loci. A *cis*-eQTL represents a genetic perturbation in a gene (between mouse strains *C57BL/6J* and *C3H/HeJ*) that leads to a change in its transcript levels. Due to the density of our genetic map (~1.5 cM), we defined the eQTL to be *cis-*acting if the physical location of the gene was within 5 megabases (Mb) of its eQTL location. The complete list of *cis-*eQTLs (logarithm of the odds score [LOD] > 4.3) for the Blue module corresponding to these mQTL regions is given in Table 1. For the Blue module mQTL, we found a total of 30 *cis*-eQTLs. Of particular interest are *Cyp2c39, Cyp2c40,* and *Cyp2c37,* which we have previously reported as candidate genes associated with weight in rodents using a different but related method in an independent F2 mouse cross [26].

We then explored whether any of the physiological trait QTL overlap with the physical location of their corresponding mQTL. First, we mapped the trait with the highest module significance score for the Blue module (i.e., body weight). The QTL curve for the single marker genome scan is shown in Figure 3. This analysis revealed the presence of four QTL (LOD > 3) on Chromosomes 1 (143 Mb), 5 (118 Mb), 15 (78 Mb), and 19 (50 Mb). Interestingly, the Chromosome 19 locus overlapped with the Blue mQTL locus. To explore whether there are interactions among the four loci mentioned above, we then fit a multiple QTL model. Using model selection [34,35], we found that the best fit is provided by a model that incorporates interactions between Chromosomes 1 and 15, and Chromosomes 1 and 19 (Table S3). In contrast to the additive model, which explained 34% of weight variation, this model was able to explain 45% of the variation in weight among the F2 mice.

### Integration of Genetics and Intramodular Connectivity to Explain Physiological Significance of the Module

Next, we sought to explore the relationships between the intramodular connectivity (k_me_), weight, and mQTL. k_me_ is the module eigengene-based connectivity (see the Materials and Methods section). To carry out this analysis, we defined two terms: GSweight and GSmQTL. As mentioned before, GSweight is defined as the absolute value of the correlation between gene expression profile and weight. GSmQTL is defined as a measure of gene significance for each of the SNPs underlying the mQTL. Using an additive marker coding (0,1,2) to code the three possible genotypes at each locus in the F2 mice, we defined the SNP gene significance measure of the i^th^ gene expression x(i) by the absolute value of its correlation with the SNP under consideration.


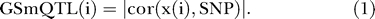


This measure is highly correlated with the traditional LOD. We prefer this correlation based SNP significance measure over the LOD because (1) this definition is consistent with our co-expression measure between two gene expression profiles and with the definition of the body weight–based gene significance measure, and (2) this definition measures the effect size of the SNP without regard to the sample size.

The mQTL-based significance measure for a particular SNP is designated “GSmQTL” followed by the chromosome number on which the corresponding SNP is located. For example, GSmQTL2 is the mQTL-based significance for the mQTL peak marker SNP on Chromosome 2. For simplicity, we make use of an additive marker coding, i.e., we assume an additive allelic effect. While there are situations when a dominant marker coding (AA→1, AB→1, and BB→0) or a recessive marker coding (AA→1, AB→0, and BB→0) is preferable, simulation studies have shown that, in general, the additive marker coding works well for a wide range of genetic models [36].

**Figure 4:**
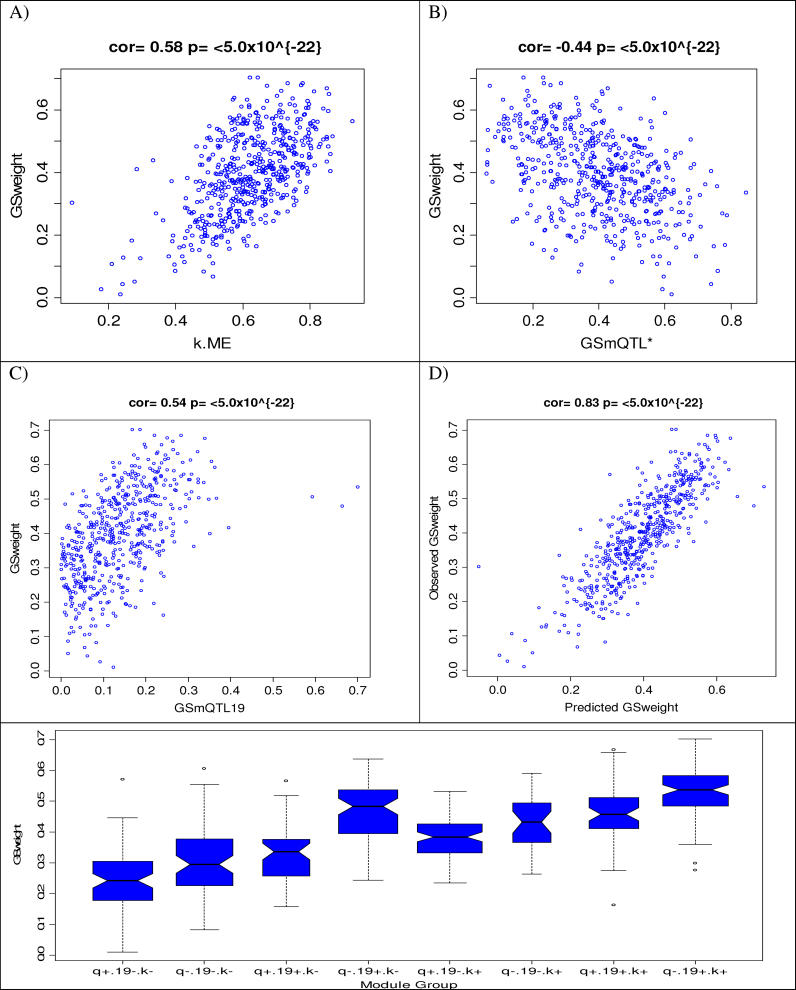
Relating the Gene Significance Measure for Weight (GSweight) to Genetic and Network Based Variables of the Blue Module (A) Scatterplot between GSweight (*y*-axis) and intramodular connectivity (k_me_) based on the module eigengene (*x*-axis). Each point corresponds to a gene in the Blue module. (B) Scatterplot between GSweight (*y*-axis) and the sum of the mQTL significance measures of Chromosomes 2, 5, and 10 (GSmQTL* = GSmQTL2 + GSmQTL5 + GSmQTL10a). (C) Scatterplot between GSweight (*y*-axis) and the mQTL significance measure of Chromosome 19 (GSmQTL19). (D) Scatterplot between GSweight (*y*-axis) and the predicted value based on a multivariable regression model involving intramodular connectivity (k_me_) and the aforementioned mQTL significance measures (GSmQTL* and GSmQTL19). The linear regression model explains 70% of the variation in GSweight. (E) Boxplot for visualizing the effect of the module based variables on GSweight. The 8 different boxplots correspond to the eight groups of genes that result by splitting the module variables by their median values. Genes with high/low GSmQTL* values are labeled by q+ and q−, respectively. Genes with high/low GSmQTL19 values are labeled by 19+ and 19−, respectively. Genes with high/low connectivity values are labeled by k+ and k−, respectively (For example, the boxplot labeled q+ 19− k− plots the GSweight distribution of the Blue module genes with a high GSmQTL*, a low GSmQTL19, and a low connectivity).

To assess the relationship between GSweight, k_me_, and GSmQTL we used a multivariate linear regression model. When we regressed GSweight on GSmQTL, we found that GSmQTL19 was positively correlated with GSweight (Figure 4) whereas GSmQTL2, GSmQTL5, and GSmQTL10a were all inversely correlated with GSweight. The latter motivated us to define a summary measure GSmQTL* = GSmQTL2 + GSmQTL5 + GSmQTL10a, which quantifies the relationship between a gene expression profile and the mQTLs on Chromosomes 2, 5, and proximal 10. By regressing GSweight on GSmQTL* and GSmQTL19, we found that 37% of the variation in the GSweight could be explained by GSmQTL on Chromosomes 2, 5, 10, and 19 (Table 2, model 1). When we regressed GSweight on connectivity (k_me_), we found that connectivity explained 34% of the variation in GSweight (Table 2, model 2). However, although GSmQTL*, GSmQTL19, and connectivity explained 19% (*r^2^* = (−0.44)^2^) (Figure 4B), 29% (Figure 4C), and 34% (Figure 4A) of the variation in GSweight, respectively, a model combining these three covariates was able to explain 70% of GSweight variation (Figure 4D; Table 2, model 3). We find that model 3 provides a significantly better fit to the data than model 1 (F test *p*-value < 2.2e^−16^). Similarly, model 3 provides a significantly better fit than model 2 (*p* < 2.2e^−16^). This analysis demonstrates that a model that integrates genetic information (SNP genotype) with network properties (intramodular connectivity) is a better predictor of the relationship between Blue module genes and weight.

**Table 2:**
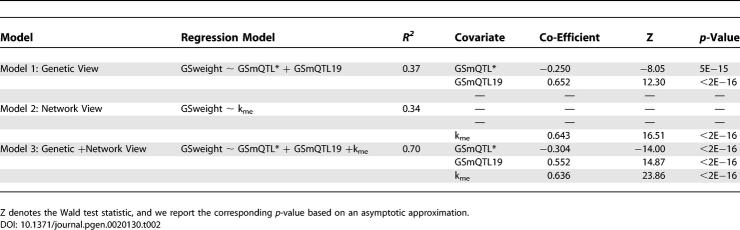
Multivariate Linear Regression Models for GSweight

To further explain the relationship between GSweight and the module variables, we visualized the distribution of GSweight in different strata using boxplots (Figure 4E). The eight different boxplots of Figure 4E correspond to the eight groups of genes that resulted by splitting the Blue module genes by their median GSmQTL*, GSmQTL19, and k_me_ values. We found that genes with high GSmQTL19, high connectivity, and low GSmQTL* had the highest GSweight (Figure 4E, labeled “q− 19+ k+”). This indicates that genes with strong linkage to the Chromosome 19 locus, absence of linkage to the SNPs described on Chromosomes 2, 5, and 10, and high connectivity have the highest absolute correlation with weight. Detailed information regarding the Blue module genes and the multivariate regression analyses can be found in Protocol S3.

## Discussion

Network approaches provide means to bridge the gap from individual genes to systems biology [8]. Highly connected “hub” nodes are of special interest because they are the backbones of the scale-free network architecture. This architecture confers resistance to change and robustness to the entire network [37]. In the co-expression network presented here, we find that the gene expression levels of hub genes are less variable (lower variance) than other, less-connected, nodes (Figure S3) across all mice. This is consistent with the idea that the network's most highly connected hubs are resilient to large genetic background variations since they are vital for core biological functions [8].

Modularity is an innate characteristic of many large networks [38] including gene co-expression networks [39]. Modules, or groups of highly correlated genes, could be a result of transcriptional co-activation (gene activation or gene repression), or the co-regulation of mRNA stability, or a combination of these, resulting in a complex genetic network of closely related genes coordinately operating to accomplish a function or a group of related functions. It is important to note that the gene modules reported here are not the only existing modules; but rather they are those most perturbed in the F2 cross examined. We find that some co-expression network modules are enriched with genes belonging to distinct cellular compartments. This finding is consistent with the study by Giot et al. [12] showing that in the *Drosophila* protein–protein interaction network, proteins within the same compartment tend to be more highly connected [12]. This also provides supporting evidence that the information extracted from the co-expression network is in concordance with the protein interaction network [40].

We show that some of the modules are directly related to physiological traits. This relationship is also evident when one examines the list of genes within each module. For example, the Blue module, which we found to be highly associated with obesity and weight, contains two key regulators of adipogenesis, namely *PPAR-γ* [41] and *BMP2* [42]. In addition, the Blue module contains genes such as *Cyp2c37, Cyp2c40, Cyp2d10, Cyp4b1,* and *Ehhadh* that are either present in the KEGG “Fatty acid metabolism” pathway or have been previously associated with adiposity [26].

An important aspect of network analysis it to study the intramodular structure and pairwise interactions between genes across multiple conditions. For example, it could be interesting to determine which pairwise interactions are tissue or sex specific. Although a detailed analysis is beyond the scope of this article, we provide a glimpse of such an analysis in Figure S4.

The “genetics of gene expression” approach has been fruitful in identifying genetic regulatory loci related to complex traits [3,6,26]. Here we show that integration of genetics and network analysis is a powerful approach to identify key regulatory loci controlling the co-expression network modules. We show that there exist several such mQTL across the genome. For the Blue module genes, we found an example of an mQTL on Chromosome 19 that coincided with the QTL of mouse weight. We hypothesize that the underlying gene or genes within such coinciding loci control the variation of both the trait and gene expression of the genes comprising the respective module. In contrast, loci that do not overlap with the trait QTL may represent novel loci that indirectly affect these traits through the underlying co-expression network.

Our unsupervised module detection method identifies a weight-related network module. In that module, genes with high intramodular connectivity tend to have a high absolute correlation with body weight (GSweight). Importantly, the availability of genetic marker data greatly enhances our understanding of the relationship between connectivity and GSweight. Two novel concepts enabled us to integrate the gene co-expression network analysis with the genetic analysis: the use of intramodular connectivity as a covariate characterizing a particular gene, and the use of the correlation of gene expression with SNP alleles at mQTL (GSmQTL) as a covariate to characterize a particular gene.

We found that the most significant predictor for the weight-based gene significance measure (GSweight) was the intramodular connectivity (k_me_). However, several of the mQTL-based gene significance measures (GSmQTL19, GSmQTL2, GSmQTL5, and GSmQTL10a) were highly significant independent predictors as well. The fact that the mQTL-based significance measures were independent of connectivity could also be seen from the weak correlations between k_me_ and GSmQTL19 (*r* = 0.083), GSmQTL2 (*r* = 0.15), GSmQTL5 (*r* = −0.071), and GSmQTL10 (*r* = 0.050). The multivariate regression models in Table 2 highlight the value of taking a network perspective. Model 3 integrates co-expression network concepts (connectivity) and genetic marker information (GSmQTL) to explain 70% of the variation in GSweight (Figure 4D). This compares favorably to the univariate model that uses only connectivity (*R^2^* = 0.34) or a model that uses only the genetic marker information (*R^2^* = 0.37). Integrating gene co-expression networks with genetic marker information allows one to understand what factors influence the relationship between gene expression and weight.

In summary, we report the construction and characterization of a gene co-expression network in mice. We demonstrate the inherent modularity of the network and show that these gene modules have biologically meaningful functions, including significant correlations with physiological traits. In addition, we show that the highly connected hub genes of a module have higher gene significance relationships to the complex trait. We also demonstrate that these gene modules are largely coordinately regulated by a few key loci on the genome. Lastly, when we integrate the genetic information at these loci with the connectivity information from our network analysis, we are able to explain 70% of the relationship between a gene and mouse weight. Our multivariate regression model represents a novel method for characterizing genes that are correlated with a complex trait, and highlights the importance of the systems biology approach to study complex traits.

## Materials and Methods

### Animal husbandry and physiological trait measurements.

*C57BL/6J apoE null (B6.apoE^−/−^)* mice were purchased from the Jackson Laboratory (Bar Harbor, Maine, United States) and *C3H/HeJ apoE null (C3H.apoE^−/−^)* mice were bred by backcrossing *B6.apoE^−/−^* to *C3H/HeJ* for ten generations with selection at each generation for the targeted *ApoE^−/−^* alleles on Chromosome 7. All mice were fed ad libidum and maintained on a 12-h light/dark cycle. F2 mice were generated by crossing *B6.apoE^−/−^* with *C3H.apoE^−/−^* and subsequently intercrossing the F1 mice. F2 mice were fed Purina Chow (Ralston-Purina Co., St. Louis, Missouri, United States) containing 4% fat until 8 wk of age, and then fed a Western diet (Teklad 88137; Harlan Teklad, Madison, Wisconsin, United States) containing 42% fat and 0.15% cholesterol for 16 wk until they were sacrificed at 24 wk of age. Mice were fasted for 4 h and anesthetized by exposure to isoflurane before the blood was collected from the retro-orbital sinus and the mice were sacrificed.

At the time of euthanasia, all mice were weighed and measured from the tip of the nose to the anus. Fat depots, plasma lipids (free fatty acids and triglycerides), plasma high-density liporprotein (HDL) cholesterol and total cholesterol, and plasma insulin levels were measured as previously described [43]. Very low-density lipoprotein (VLDL)/LDL cholesterol levels were calculated by subtracting HDL cholesterol from total cholesterol levels. Plasma glucose concentrations were measured using a glucose kit (#315–100; Sigma, St. Louis, Missouri, United States). Plasma leptin, adiponectin, and MCP-1 levels were measured using mouse enzyme-linked immunoabsorbent (ELISA) kits (#MOB00, #MRP300, and MJE00; R&D Systems, Minneapolis, Minnesota, United States).

### Genotyping.

Genomic DNA was isolated from kidney by phenol-chloroform extraction. An examination of existing databases identified over 1,300 SNPs that showed variation between the *C57BL/6J* and *C3H/HeJ* strains, and a complete linkage map for all 19 autosomes was constructed using 1,065 of these SNPs at an average density of 1.5 cM. Genotyping was conducted by ParAllele (Affymetrix, Santa Clara, California, United States) using the molecular-inversion probe (MIB) multiplex [44].

### Microarray analysis.

RNA preparation and array hybridizations were performed at Rosetta Inpharmatics (Seattle, Washington, United States). The custom ink-jet microarrays used in this study (Agilent Technologies [Palo Alto, California, United States], previously described [3,45]) contain 2,186 control probes and 23,574 non-control oligonucleotides extracted from mouse Unigene clusters and combined with RefSeq sequences and RIKEN full-length clones. Mouse livers were homogenized and total RNA extracted using Trizol reagent (Invitrogen, Carlsbad, California, United States) according to manufacturer's protocol. Three μg of total RNA was reverse transcribed and labeled with either Cy3 or Cy5 fluorochromes. Purified Cy3 or Cy5 complementary RNA was hybridized to at least two microarray slides with fluor reversal for 24 h in a hybridization chamber, washed, and scanned using a laser confocal scanner. Arrays were quantified on the basis of spot intensity relative to background, adjusted for experimental variation between arrays using average intensity over multiple channels, and fit to an error model to determine significance (type I error). Gene expression is reported as the ratio of the mean log_10_ intensity (mlratio) relative to the pool derived from 150 mice randomly selected from the F2 population.

### Microarray data reduction.

In order to minimize noise in the gene expression dataset, several data-filtering steps were taken. First, preliminary evidence showed major differences in gene expression levels between sexes among the F2 mice used, and therefore only female mice were used for network construction. The construction and comparison of the male network will be reported elsewhere. Only those mice with complete phenotype, genotype, and array data were used. This gave a final experimental sample of 135 female mice used for network construction. To reduce the computational burden and to possibly enhance the signal in our data, we used only the 8,000 most-varying female liver genes in our preliminary network construction. For module detection, we limited our analysis to the 3,600 most-connected genes because our module construction method and visualization tools cannot handle larger datasets at this point. By definition, module genes are highly connected with the genes of their module (i.e., module genes tend to have relatively high connectivity). Thus, for the purpose of module detection, restricting the analysis to the most-connected genes should not lead to major information loss. Since the network nodes in our analysis correspond to genes as opposed to probesets, we eliminated multiple probes with similar expression patterns for the same gene. Specifically, the 3,600 genes were examined, and where appropriate, gene isoforms and genes containing duplicate probes were excluded by using only those with the highest expression among the redundant transcripts. This final filtering step yielded a count of 3,421 genes for the experimental network construction.

### Weighted gene co-expression network construction.

Constructing a weighted co-expression network is critical for identifying modules and for defining the intramodular connectivity. In co-expression networks, nodes correspond to genes, and connection strengths are determined by the pairwise correlations between expression profiles. In contrast to unweighted networks, weighted networks use soft thresholding of the Pearson correlation matrix for determining the connection strengths between two genes. Soft thresholding of the Pearson correlation preserves the continuous nature of the gene co-expression information, and leads to results that are highly robust with respect to the weighted network construction method [27].

The theory of the network construction algorithm is described in detail elsewhere [27]. Briefly, a gene co-expression similarity measure (absolute value of the Pearson product moment correlation) was used to relate every pairwise gene–gene relationship. An adjacency matrix was then constructed using a “soft” power adjacency function *a_ij_* = |*cor(x_i_, x_j_)| ^β^* where the absolute value of the Pearson correlation measures gene is the co-expression similarity, and *a_ij_* represents the resulting adjacency that measures the connection strengths. The network connectivity (k_all_) of the i^th^ gene is the sum of the connection strengths with the other genes, i.e., 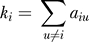 . This summation performed over all genes in a particular module is the intramodular connectivity (k_in_). The network satisfies scale-free topology if the connectivity distribution of the nodes follows an inverse power law, (frequency of connectivity p(k) follows an approximate inverse power law in k, i.e., p(k) ~ k^−γ^). We chose a power of *β* = 6 based on the scale-free topology criterion [27]. This criterion says that the power parameter, *β,* is the lowest integer such that the resulting network satisfies approximate scale-free topology (linear model fitting index *R^2^* of the regression line between log(p(k)) and log(k) is larger than 0.8). This criterion uses the fact that gene co-expression networks have been found to satisfy *approximate* scale-free topology [8,46–48]. Since we are using a weighted network as opposed to an unweighted network, the biological findings are highly robust with respect to the choice of this power. Many co-expression networks satisfy the scale-free property only approximately. Figure S5 shows that the connectivity distribution p(k) is better modeled using an exponentially truncated power law p(k) ~ k^−γ^ exp(−α k) [49]. In practice, we find that the two parameters α and γ provide too much flexibility in curve fitting. The truncated exponential model fitting index *R^2^* tends to be high irrespective of the adjacency function parameter. For this reason, we focus on the scale-free topology fitting index in our scale-free topology criterion. Exploring the use of the truncated exponential fitting index is beyond the scope of this article.

### Network module identification.

The modules in our weighted gene co-expression network are groups of highly correlated genes. In network terminology, modules are groups of genes with similar patterns of connection strengths with all other genes of the network. Modules are sets of genes with high “topological overlap” [27,50]. To calculate the topological overlap for a pair of genes, we compare them in terms of their connection strengths with all other genes in the network. A pair of genes is said to have high topological overlap if they are both strongly connected to the same group of genes. The use of topological overlap thus serves as a filter to exclude very weak connections during network construction. Specifically the topological overlap between genes *i* and *j* is given by

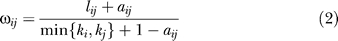
where 
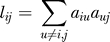
 denotes the number of nodes to which both *i* and *j* are connected, and *u* indexes the nodes of the network. The topological overlap matrix (TOM) is given by Ω = [ω_ij_]. ω_ij_ is a number between 0 and 1 and is symmetric (i.e, ω_ij_ = ω_ji_). Module identification was based on using the topological overlap matrix, Ω = [ω_ij_] in conjunction with average linkage hierarchical clustering. Since hierarchical clustering takes a *dis*similarity measure as input, we defined a topological overlap–based dissimilarity measure as follows: 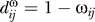 . Modules corresponded to branches of the resulting hierarchical clustering tree (dendrogram). We used a dynamic cut-tree algorithm for selection branches of the hierarchical clustering dendrogram (the details of the algorithm can be found at the following link: http://www.genetics.ucla.edu/labs/horvath/binzhang/DynamicTreeCut). The algorithm takes into account an essential feature of cluster occurrence and makes use of the internal structure in a dendrogram. Specifically, the algorithm is based on an adaptive process of cluster decomposition and combination, and the process is iterated until the number of clusters becomes stable. Although our network and module construction method has been used in other applications [27,33], no claim is made that our module construction method is optimal. The findings of a simulation study that show the efficacy of our module detection method can be found on our Web page: http://www.genetics.ucla.edu/labs/horvath/CoexpressionNetwork/MouseWeight.

Each module represents a group of genes with similar expression profiles across the samples and the expression profile pattern is distinct from those of the other modules. In each case, the modules were then assessed for enrichment in ontology terms based on Fisher's exact test.

### Statistical analysis of module significance measure and eQTL clustering.

To assign a *p*-value to the module significance measure, we made use of the Fisher transformation as implemented in the R function “cor.test”. Since the claims regarding the relationship between module genes and physiologic traits is fundamental to many subsequent analyses, we used the conservative Bonferroni correction for multiple comparisons which entails multiplying the uncorrected *p*-value by the number of tests. A less-conservative alternative would be to estimate the false discovery rate, but care needs to be taken because the tests may be highly correlated.

### Module connectivity and mQTL used in regression analysis.

For our multivariate regression analyses, we find it expedient to sum up the gene expression profiles of an entire module by one idealized gene expression profile. Toward this end we make use of the concept of a module eigengene. For a detailed theoretical discussion see http://www.genetics.ucla.edu/labs/horvath/ModuleConformity.

Briefly, to compute the module eigengene, one decomposes the standardized gene expression profile of each module via the singular value decomposition (X = UDV^T^). The first column V_1_ of V corresponds to the module eigengene. The singular value decomposition is closely related to principal component analysis, we denote V_1_ by PC1 in the text. The module eigengene gives rise to a measure of module centrality (k_me_) as follows: k_me_(i) = |cor(x(i),PC1)|. We will refer to k_me_ as module eigengene-based connectivity. The module eigengene-based connectivity measure (k_me_) is highly correlated (*r* = 0.86) with the standard intramodular connectivity measure (k_in_). k_me_ was used only in our regression analysis because it is slightly more informative in our regression models. For all other applications, k_in_ was utilized.

The SNPs used in the multivariate linear regression analysis were chosen based on two criteria: (1) They had to be peak markers at the Blue module mQTL, and (2) they had to be significant predictors of weight (*p* < 0.05) in the linear model in which weight was regressed on all nine mQTL SNPs. These two criteria resulted in Chromosomes 2, 5, 10a, and 19 mQTL SNPs being included in the multivariate linear regression analysis.

### EASE Analysis.

The Expression Analysis Systemic Explorer (EASE) software [29] was downloaded from the Database for Annotation, Visualization, and Integrated Discovery (DAVID) [51] http://david.niaid.nih.gov/david/ease.htm. As detailed in Protocol S2, each module was analyzed separately for pathway enrichment. We also report corresponding pathway plots. In Table 1 of Protocol S2, we report Fisher exact *p*-values and several multiple testing corrected *p*-values (Bonferroni, Benjamini, and Hochberg).

### Other statistical methods and software.

Linear regression modeling was performed using the R function “lm” (25). The percent of variance explained by the linear model was determined using the unadjusted *R^2^* value of the model. In the text, we also made use of the well-known statistical result that the variance explained by a univariate linear model involving a single regressor *x* and an outcome *y* equals the square of the correlation between *x* and *y*.

The boxplots of Figure 4E visualize the distribution of GSweight for different groups of genes. A boxplot consists of the most extreme values (the whiskers) in the dataset (maximum and minimum values), the lower and upper quartiles (lower and upper boundary of the box), and the median value (horizontal line inside the notch). A notch is drawn in each side of the box. If the notches of two plots do not overlap, the two medians differ significantly. Single-point QTL analyses used the “scanone” function of the rQTL package.

To compare nested linear models, we used the “anova” function in R. The fitqtl command in qtl package [35] in R software was used to carry out the model selection for weight.

### Software and data availability.

The statistical analysis was carried out with the R software (http://www.R-project.org). We provide the entire statistical code and processed data for generating the weighted gene co-expression network results. Thus, the reader is able to reproduce our findings. The document also serves as a tutorial to weighted gene co-expression network analysis. This tutorial and the data files can be found at the following Web page: http://www.genetics.ucla.edu/labs/horvath/CoexpressionNetwork/MouseWeight.

## Supporting Information

Figure S1 MS Score for All Modules Corresponding to Each Trait Bars representing MS are colored according to the designated color of their corresponding module. An MS score of 0.30 (red horizontal line) is significant at *p* = 0.05 after a Bonferroni correction for multiple comparisons.
(442 KB PDF)

Figure S2 Connectivity and Gene Significance in the Blue Module (A) Scatter plot representing the relationship of intramodular connectivity (k_in_) with gene significance for weight in the Blue module in different tissue/gender combinations. The corresponding tissue/gender combination is stated on the *x*-axis. Gene significance is defined as the correlation coefficient for each gene with the physiological trait of interest. (B) Scatter plot between female liver connectivity (k_in_) in the Blue module (*x*-axis) and intramodular connectivities in other tissue/gender combinations. The corresponding tissue/gender combination is stated on the *y*-axis. The correlations show that the intramodular connectivities of the Blue module genes are not preserved. This may indicate that the Blue module is liver specific.
(406 KB PDF)

Figure S3 The Relationship between Connectivity and Gene Expression in the Blue Module and the Complete Network (A) Plots representing the relationship between connectivity (k_in_) with mean expression level and gene expression variance of the Blue module. (B) Plots representing the relationship between connectivity (k_all_) and gene expression mean and variance for all genes in the entire co-expression network.
(323 KB PDF)

Figure S4 Visualizing Connection Patterns of Five Hub Genes across Different Gender/Tissue Combinations The Figure shows focuses on the five most-connected hub genes in the female liver tissue and their strongest connections with the Blue module genes. The strongest connections were defined as the upper 10% of pairwise topological overlap. There is some suggestive evidence for sex differences in *Ppic* connectivity in liver and adipose tissue. *Anxa5* appears to be highly connected in liver but less so in adipose or muscle. A detailed and more rigorous analysis is beyond the scope of our article.
(1.5 MB PDF)

Figure S5 Log/Log Plot Representing the Connectivity Distribution of the Network The black line is the best linear fit to the log k/log p(k) plot, where k is connectivity. This fit is representative of a power-law distribution indicative of a scale-free topology. The red line is representative of a log/log plot fitting a model representative of an exponentially truncated power law p(k) ~ k^−γ^ exp(−α k). Both models have previously been described as characteristic of naturally occurring networks.
(4 KB PDF)

Protocol S1 Functional Enrichment (EASE) Analysis (1.6 MB DOC)

Protocol S2 Gene Information, Network Connectivity, and Gene Expression Data (8.9 MB XLS)

Protocol S3 Data for Blue Module Regression Analysis (245 KB XLS)

Table S1 Characteristics of the Female Liver Gene Co-Expression Modules (32 KB DOC)

Table S2 Summary of Blue Module Hub Genes and Their Properties (158 KB DOC)

Table S3 Regressing Body Weight on Multiple mQTLs (SNPs) of the Blue Module (25 KB DOC)

### Accession Numbers

The Gene Expression Omnibus (GEO; http://www.ncbi.nlm.nih.gov/geo) accession number for this entire liver gene expression dataset is GSE2814.

## Abbreviations

eQTL: expression quantitative trait locus
GSmQTL: mQTL-based gene significance measure
GSweight: gene significance for weight
KEGG: Kyoto Encyclopedia of Genes and Genomes
k_in_: intramodular connectivity
LOD: logarithm of the odds ratio
Mb: megabases
mQTL: module quantitative trait locus
MS: module significance
QTL: quantitative trait locus
SNP: single nucleotide polymorphism
