# Supplementary material for: Integrating Genetic and Network Analysis to Characterize Genes Related to Mouse Weight
Source: PLoS Genet. 2006 Aug 18;2(8):e130. doi: 10.1371/journal.pgen.0020130 (PMC1550283; doi:10.1371/journal.pgen.0020130)
Supplement: Supplementary file 1 [file pgen.0020130.sg001.pdf]

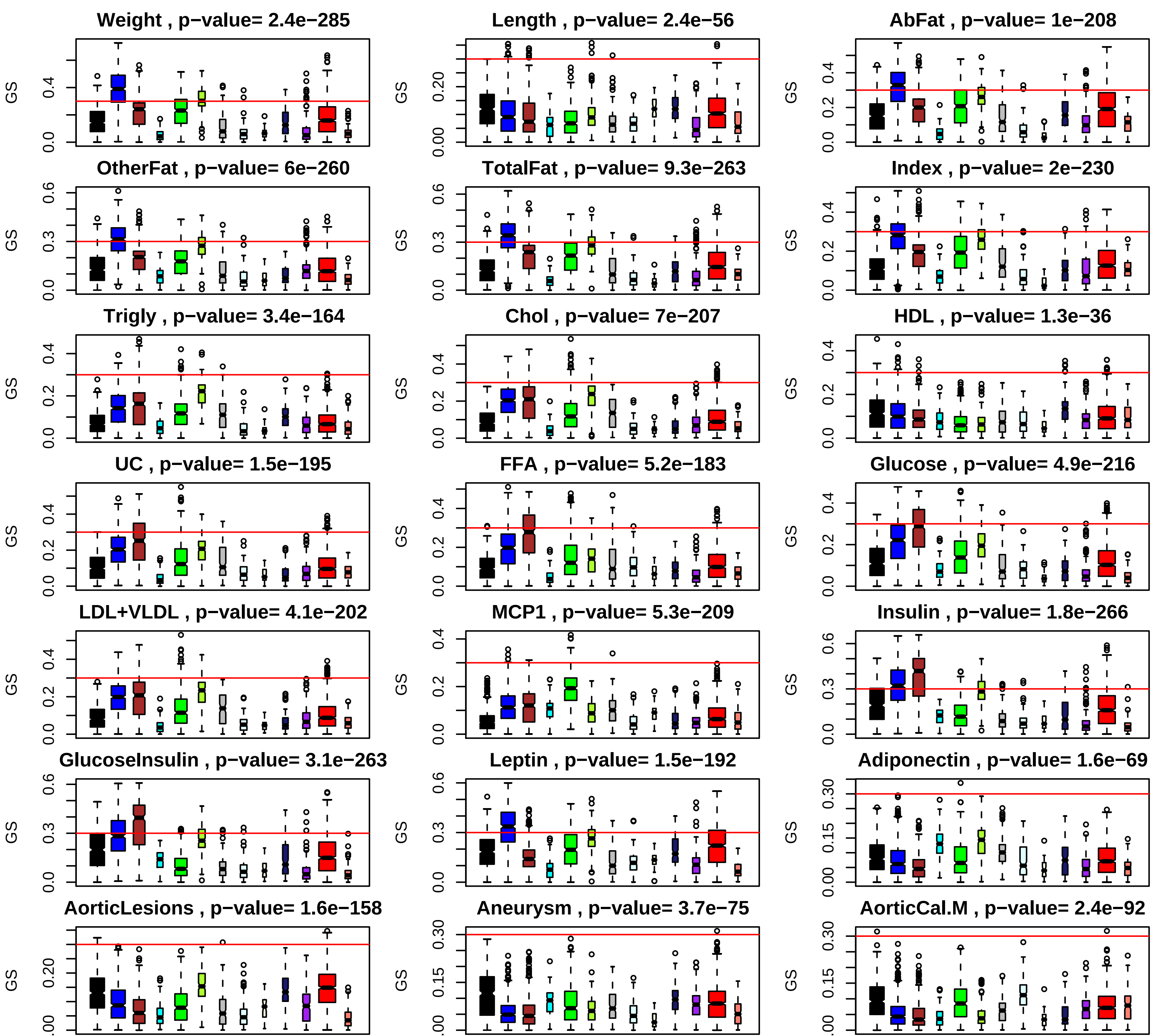

Weight , p−value= 2.4e−285
Length , p−value= 2.4e−56
AbFat , p−value= 1e−208
OtherFat , p−value= 6e−260
TotalFat , p−value= 9.3e−263
Index , p−value= 2e−230
Trigly , p−value= 3.4e−164
Chol , p−value= 7e−207
HDL , p−value= 1.3e−36
UC , p−value= 1.5e−195
FFA , p−value= 5.2e−183
Glucose , p−value= 4.9e−216
LDL+VLDL , p−value= 4.1e−202
MCP1 , p−value= 5.3e−209
Insulin , p−value= 1.8e−266
GlucoseInsulin , p−value= 3.1e−263
Leptin , p−value= 1.5e−192
Adiponectin , p−value= 1.6e−69
AorticLesions , p−value= 1.6e−158
Aneurysm , p−value= 3.7e−75
AorticCal.M , p−value= 2.4e−92
GS
