# Supplementary material for: Integrating Genetic and Network Analysis to Characterize Genes Related to Mouse Weight
Source: PLoS Genet. 2006 Aug 18;2(8):e130. doi: 10.1371/journal.pgen.0020130 (PMC1550283; doi:10.1371/journal.pgen.0020130)

A) GSweight versus intramodular connectivity

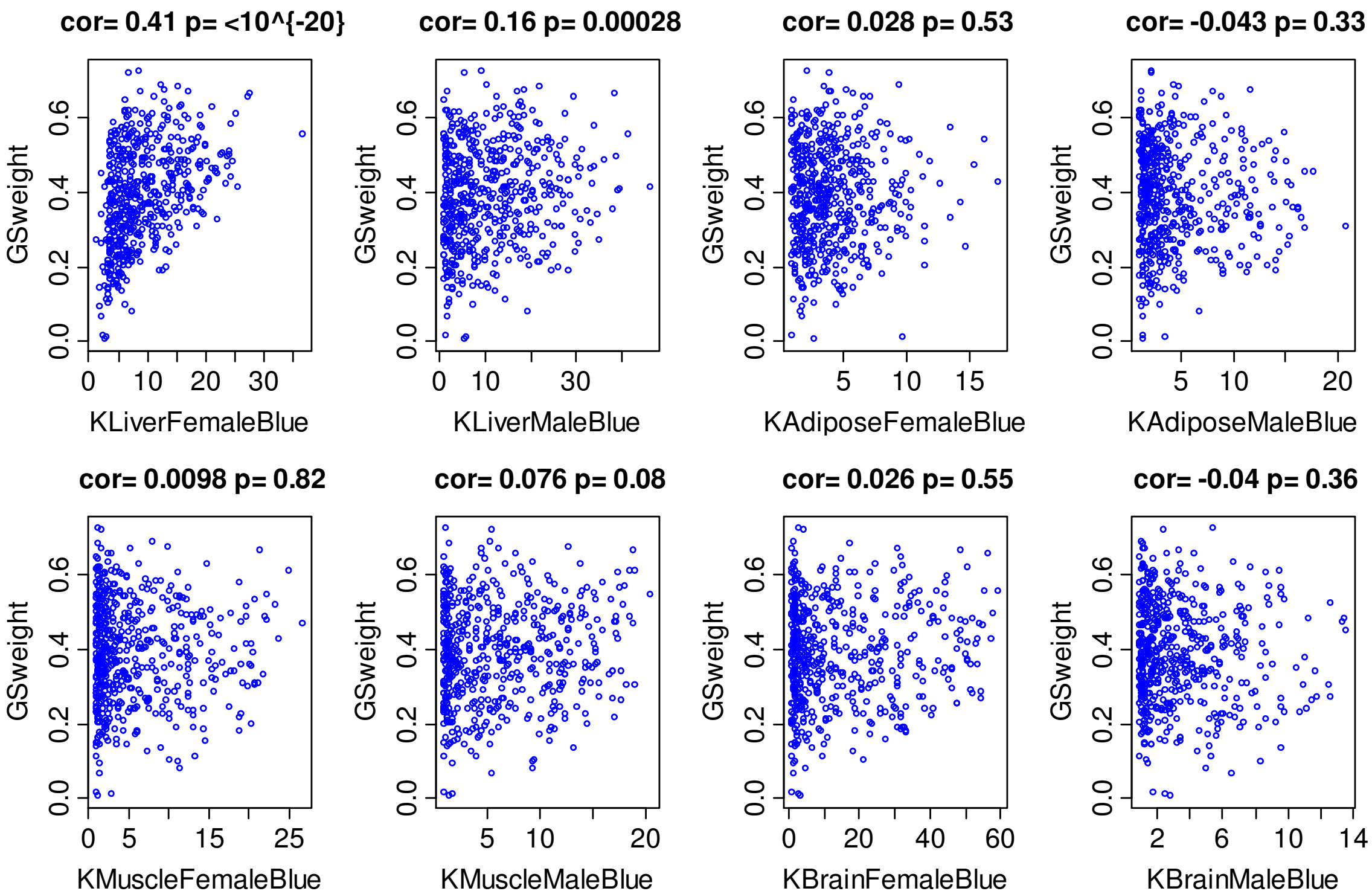

B) Connectivity versus intramodular connectivity in the female mouse liver network

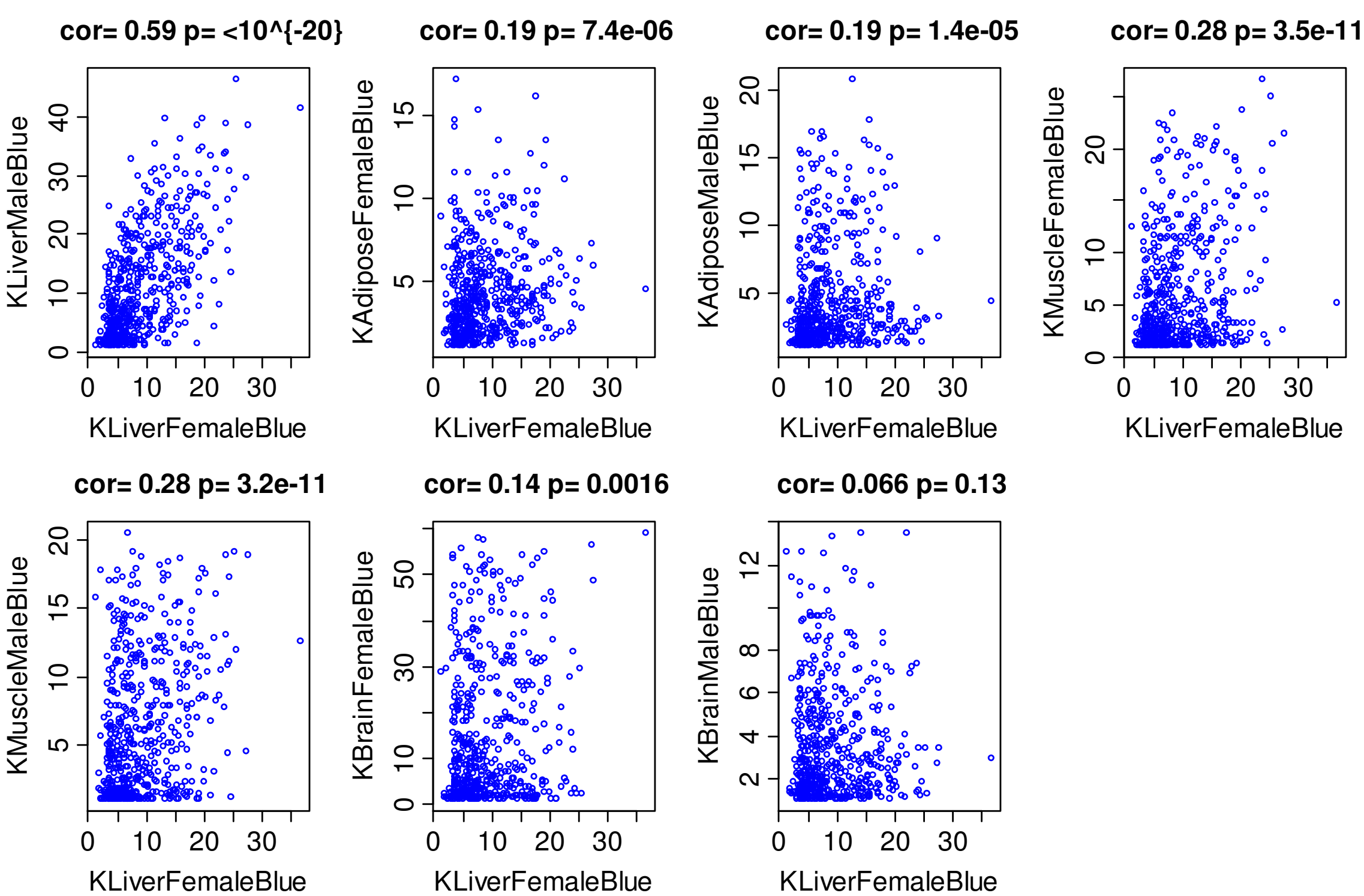

Supplement: Supplementary file 2 [file pgen.0020130.sg002.pdf]
