# Supplementary material for: Integrating Genetic and Network Analysis to Characterize Genes Related to Mouse Weight
Source: PLoS Genet. 2006 Aug 18;2(8):e130. doi: 10.1371/journal.pgen.0020130 (PMC1550283; doi:10.1371/journal.pgen.0020130)
Supplement: Supplementary file 3 [file pgen.0020130.sg003.pdf]

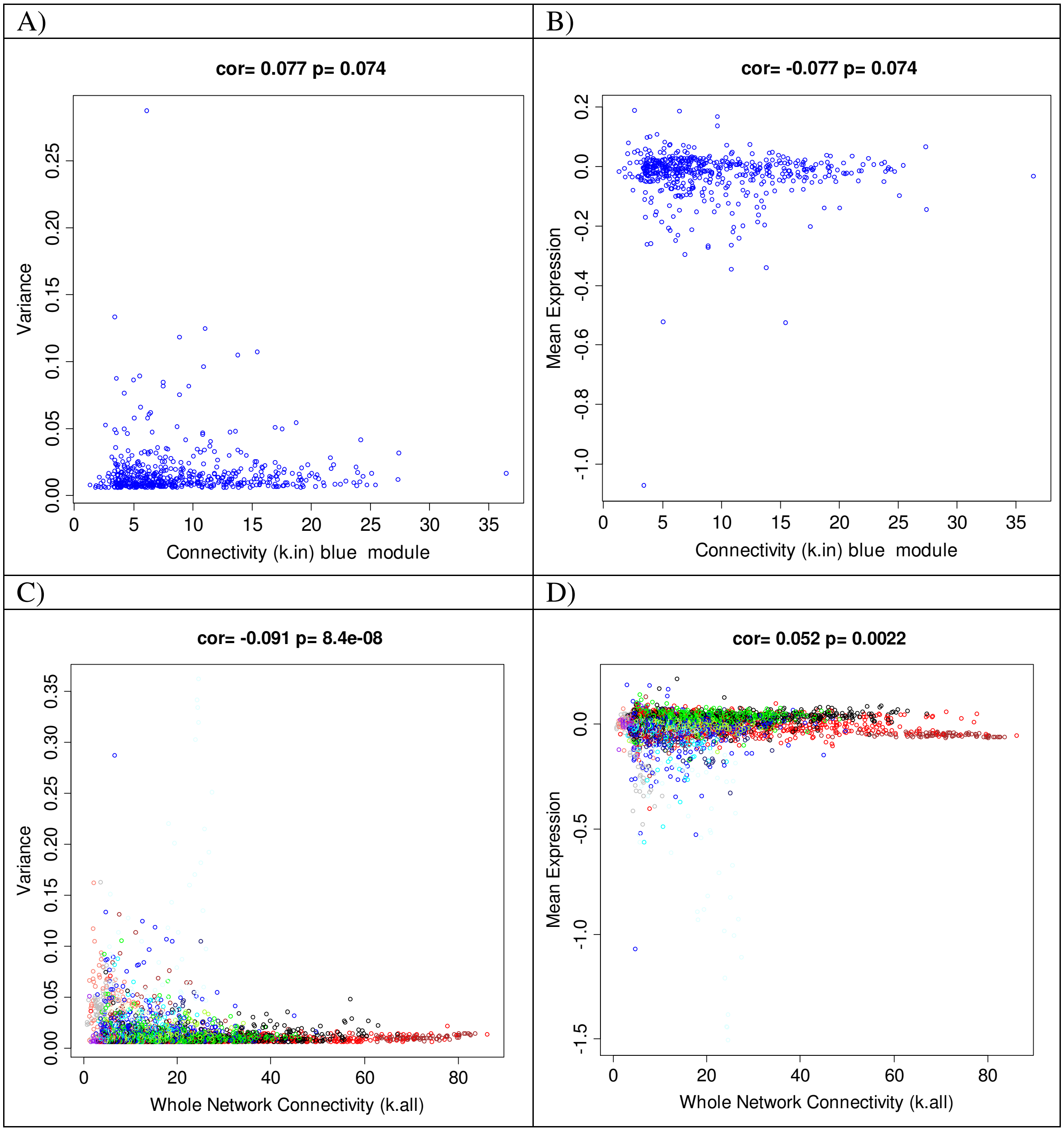
A)
cor= 0.077 p= 0.074
Variance
0.00
0.05
0.10
0.15
0.20
0.25
0
5
10
15
20
25
30
35
Connectivity (k.in) blue module
B)
cor= -0.077 p= 0.074
Mean Expression
-1.0
-0.8
-0.6
-0.4
-0.2
0.0
0.2
0
5
10
15
20
25
30
35
Connectivity (k.in) blue module
C)
cor= -0.091 p= 8.4e-08
Variance
0.00
0.05
0.10
0.15
0.20
0.25
0.30
0.35
0
20
40
60
80
Whole Network Connectivity (k.all)
D)
cor= 0.052 p= 0.0022
Mean Expression
-1.5
-1.0
-0.5
0.0
0
20
40
60
80
Whole Network Connectivity (k.all)
