# Supplementary material for: Integrating Genetic and Network Analysis to Characterize Genes Related to Mouse Weight
Source: PLoS Genet. 2006 Aug 18;2(8):e130. doi: 10.1371/journal.pgen.0020130 (PMC1550283; doi:10.1371/journal.pgen.0020130)
Supplement: Supplementary file 4 [file pgen.0020130.sg004.pdf]

Supplementary Figure S4.

	Female	Male
Liver	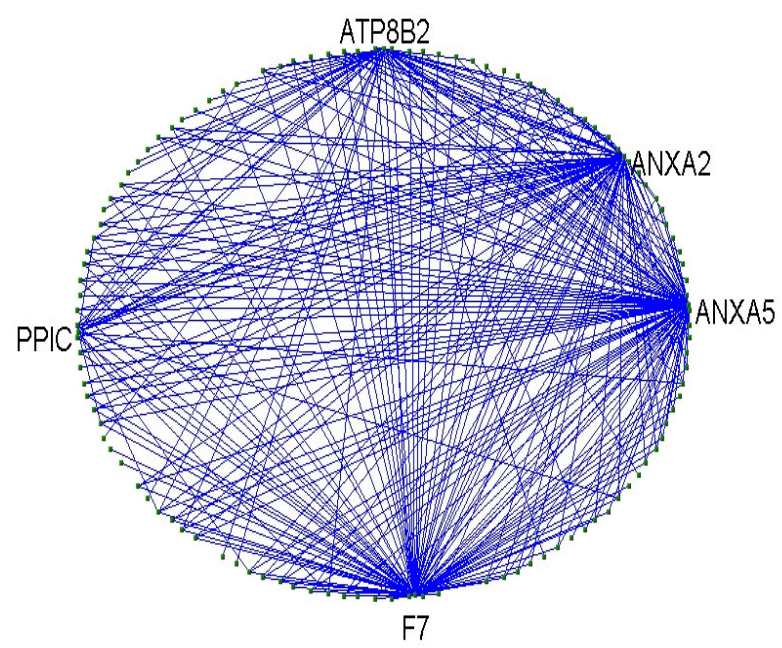	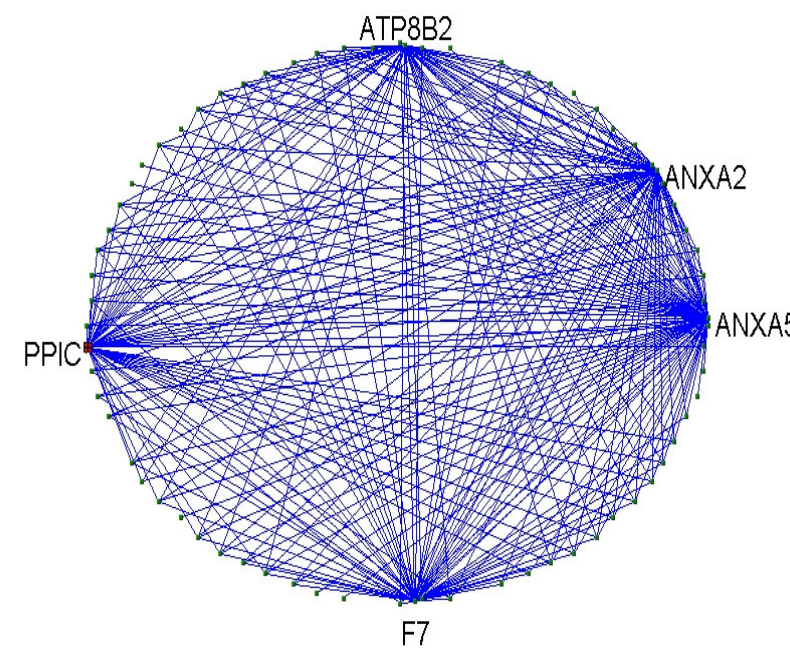
Adipose	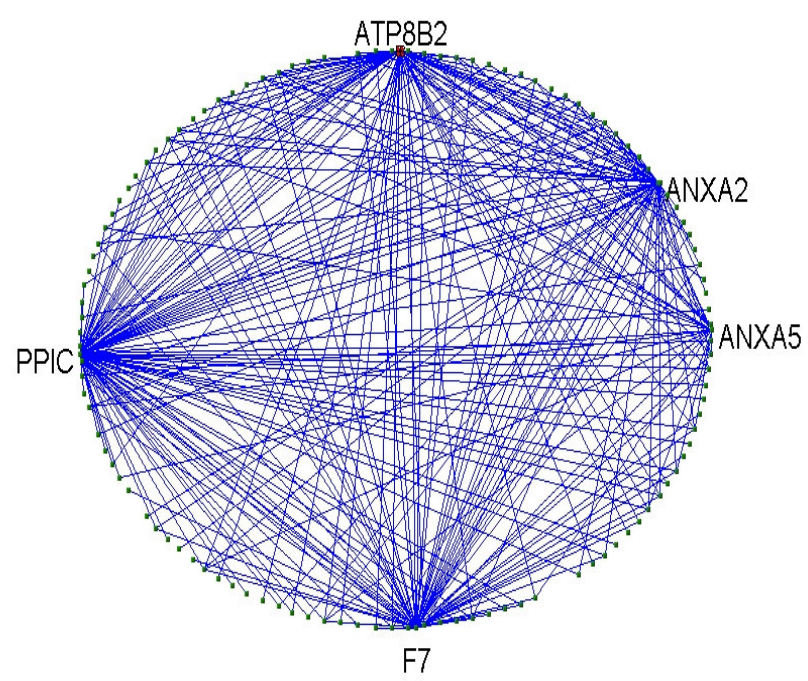	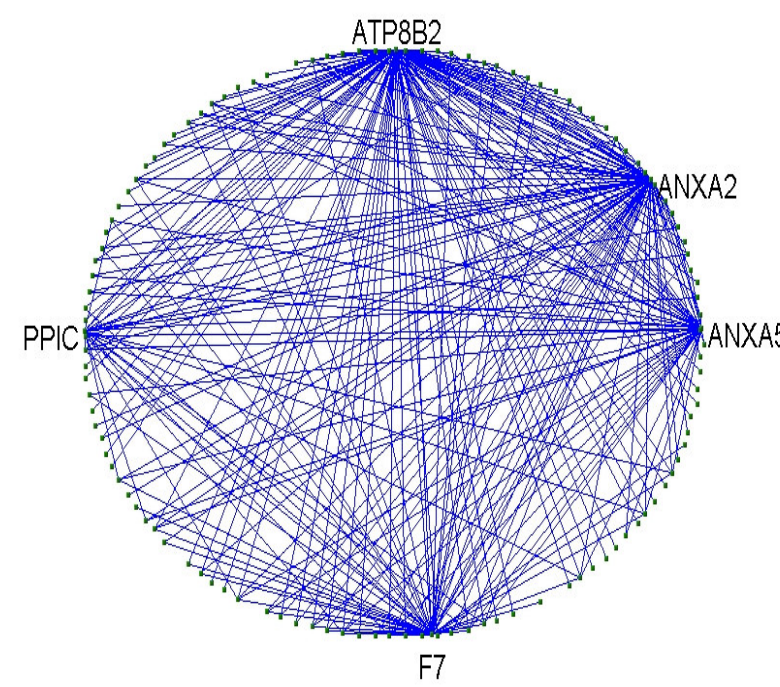
Muscle	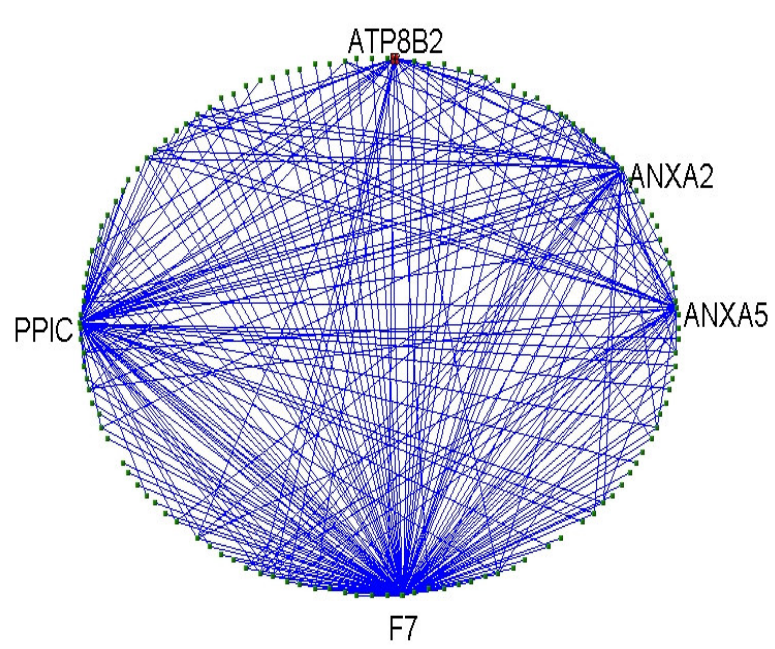	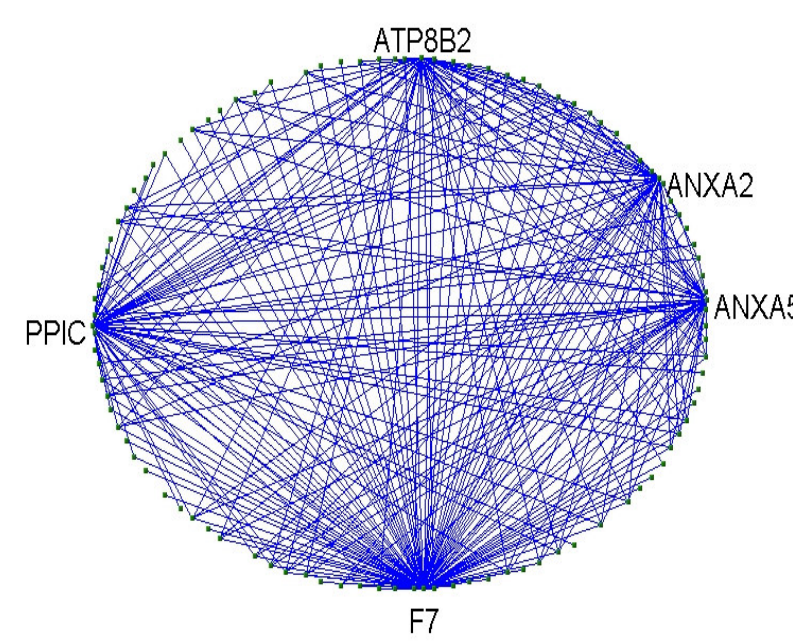
