# Supplementary material for: Integrating Genetic and Network Analysis to Characterize Genes Related to Mouse Weight
Source: PLoS Genet. 2006 Aug 18;2(8):e130. doi: 10.1371/journal.pgen.0020130 (PMC1550283; doi:10.1371/journal.pgen.0020130)
Supplement: Supplementary file 5 [file pgen.0020130.sg005.pdf]

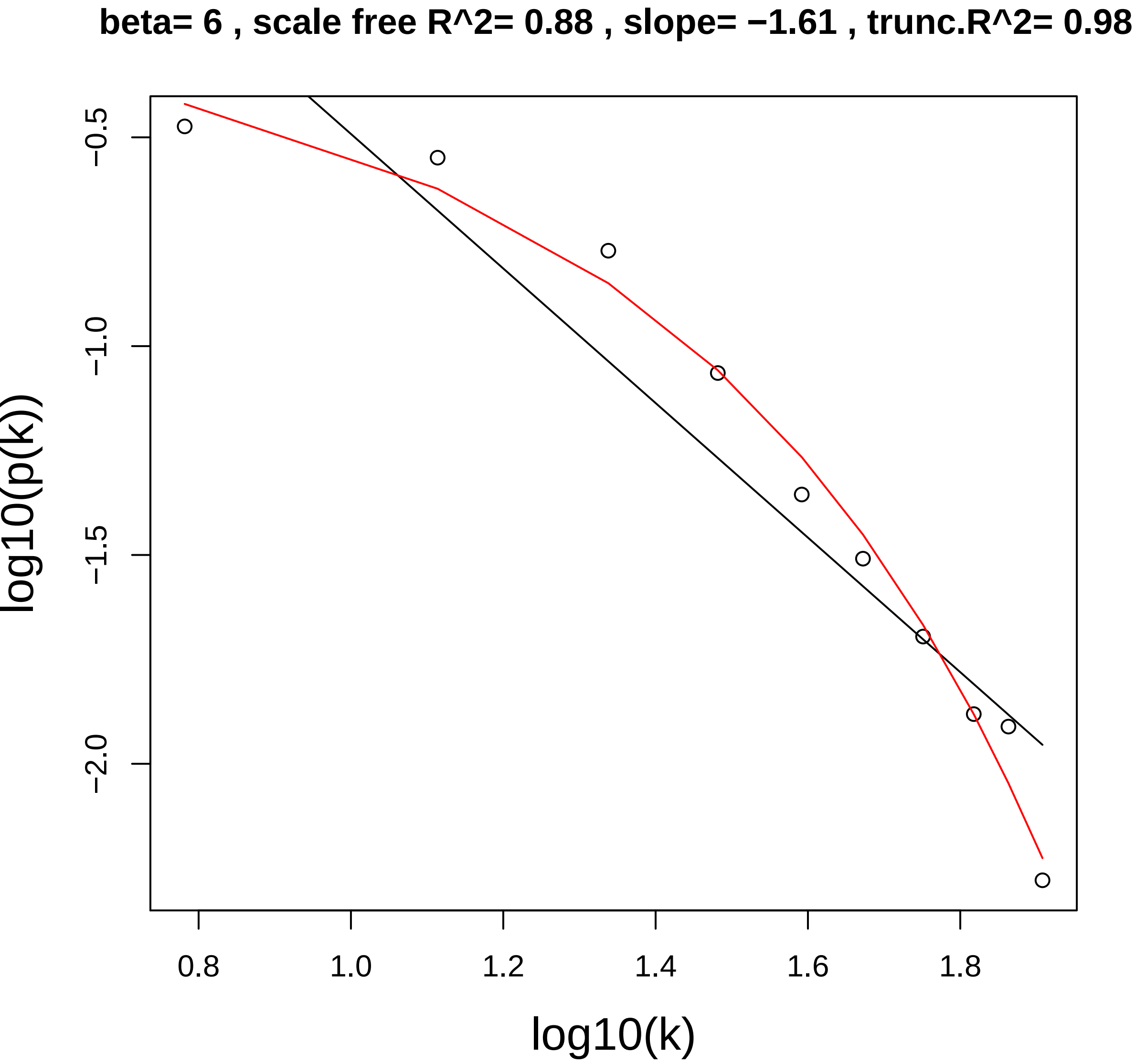
beta= 6 , scale free R^2= 0.88 , slope= −1.61 , trunc.R^2= 0.98
−0.5
−1.0
−1.5
−2.0
log10(p(k))
0.8
1.0
1.2
1.4
1.6
1.8
log10(k)
