# Supplementary material for: Integrating Genetic and Network Analysis to Characterize Genes Related to Mouse Weight
Source: PLoS Genet. 2006 Aug 18;2(8):e130. doi: 10.1371/journal.pgen.0020130 (PMC1550283; doi:10.1371/journal.pgen.0020130)
Supplement: Supplementary file 6 [file pgen.0020130.sd001.doc]

**Protocol S1:**

Here we provide details on the functional enrichment analysis of the gene modules described in the following article.

*Anatole Ghazalpour, Sudheer Doss, Bin Zhang, Susanna Wang,Christopher Plaisier, Ruth Castellanos, Alec Brozell, Eric E. Schadt, Thomas A. Drake, Aldons J. Lusis, Steve Horvath (2006) Integrating Genetic and Network Analysis to Characterize Genes Related to Mouse Weight. PloS Genetics*

In order to gain further insight into the functional significance of the gene modules, we utilized the Expression Analysis Systemic Explorer (EASE) software package [29] which conducts a pathway enrichment analysis for each module. The Expression Analysis Systemic Explorer (EASE) program was downloaded from the Database for Annotation, Visualization, and Integrated Discovery (DAVID). In particular, we used EASE to study the enrichment of pathways annotated in the Kyoto Encyclopedia of Genes and Genomes (KEGG). The details can be found in Table 1 (below), which reports the module name, KEGG pathway identifier, KEGG pathway name, p-value, and the module genes found in the respective pathway.

As primary statistic we chose Fisher’s exact test but we also report the results of the following multiple testing correction procedures: Bonferroni, Benjamini and Hochberg.

The EASE analysis for functional enrichment of modules found that seven modules were significantly enriched.

The Blue module was significantly enriched for ‘ECM-receptor interaction’ p = 2.26x10^-9^), ‘Complement and coagulation cascades’ p = 1.01x10^-6^). The other less significant pathways also showed a strong association of this module with extracellular matrix and structural functions.

The Brown module was significantly enriched for ‘Biosynthesis of steroids’, and ‘Glycolysis / Gluconeogenesis’. The ‘Biosysnthesis of steroids’ pathway p = 2.25x10^-6^) is enriched with genes that are leading to the production of cholesterol. The ‘Glycolysis / Gluconeogenesis’ pathway p = 1.52x10^-5^) function in this module may be related to pyruvate metabolism. The ‘Glycolysis / Gluconeogenesis’ pathways are enriched with genes leading to the production of pyruvate. There is in turn a link from ‘Pyruvate metabolism’ p = 0.0014) to ‘Fatty Acid Biosysnthesis’ (path 1) which can be observed in Figure 1. Both the ‘Fatty Acid Biosynthesis’ (path 1) pathway p = 0.038) and ‘Fatty Acid Biosysnthesis’ (path 2) p = 0.038) yielded p-values that were suggestive of significance. A majority of the enriched pathways in this module were related to sugar metabolism and side pathways along the route to fatty acid biosynthesis. This is consistent with our finding that this module was closely related to plasma glucose/insulin ratio in mice (supplementary Figure 2)

The Green module is significantly enriched for ‘Toll-like receptor signaling pathway’ p = 1.59x10^-8^), ‘Cytokine-cytokine receptor interaction’ p = 2.87x10^-7^), and ‘Hematopoietic cell lineage’ p = 6.57x10^-5^). The ‘Cytokine-cytokine receptor interaction’ pathway is related to ‘Hematopoietic cell differentiation’ and most of the pathways involved in this module are related to either hematopoiesis, inflammatory response, or the immune system. This is an interesting module function but not completely surprising because there are hematopoietic cells present in the liver.

The GreenYellow module is highly enriched in ‘Cell cycle’ pathway p = 6.26x10^-18^). There are 22 genes in this module that are annotated by KEGG, and 15 of these genes are in the ‘Cell cycle’ pathway. The other less significantly enriched pathways are also related in one way or another to cell cycle: ‘DNA polymerase’ p = 0.004), ‘Pyrimidine metabolism’ p = 0.016), ‘Purine metablolism’ p = 0.063). One interpretation of these enriched pathways in the same module is that this module is characteristic of actively dividing cells in the liver, possibly stem cells.

The LightYellow module was only significantly enriched for genes in the ‘MAPK signaling’ pathway p = 0.0054).

The MidnightBlue module was significantly enriched for the ‘Nitrobenzene degradation’ pathway p = 0.00071). The ‘Nitrobenzene degradation’ pathway has some of the same enzymes as ‘Androgen and estrogen metabolism’ pathway p = 0.0041) which is also suggestively enriched. The function of this pathway is somewhat complex, and in order to determine the function further analysis is necessary.

The Red module was significantly enriched for the ‘Ribosome components’ p = 0.00031). It was also suggestively enriched for the ‘Proteasome’ p = 0.0029), and ‘RNA Polymerase’ p = 0.012), which suggests the function of this module is related to transcription and translation.

**The data were input into EASE as follows:**

The module gene identfiers were copy and pasted into the “Input Genes” field and all genes were chosen as a control set (“All Genes Assayed” field). The categorical system was set to the annotations that we retrieved from KEGG.

**KEGG pathways**

Figure 1 depicts the KEGG diagrams of the significant pathways described in the text. The p-values for the corresponding pathways can be found in Table 1. The enzymatic reactions that are found in mice are highlighted by coloring the boxes green. The white boxes are not found in mice. The boxes with red outlines and red text are genes found in the module. By following the red boxes through the pathways one may gain insights regarding the module function. Boxes in green that are not highlighted in red outlines may be present in the mouse liver but may not be present in the list of filtered genes.

Table 2 (below) maps the locus link IDs to their respective KEGG Pathway ID.

**References**

[1] Hosack DA, Dennis G Jr, Sherman BT, Lane HC, Lempicki RA: Identifying biological themes within lists of genes with EASE*.Genome Biol* 2003, 4:R70.

[2] Dennis G Jr, Sherman BT, Hosack DA, Yang J, Gao W, Lane HC, Lempicki RA: DAVID: Database for Annotation, Visualization, and Integrated Discovery*. Genome Biol* 2003, 4:P3

**Table 1.** EASE analysis results. (Pathways with yellow backgrounds have p-values less than 0.05)

| **Module** | **Pathway Description** | **Uncorrected p-value** | **Bonferroni p-value** | **Hochberg p-value** | **Benjamini p-value** |
| --- | --- | --- | --- | --- | --- |
| Black | ATP synthesis | 0.00116601 | 6.65E-02 | 6.65E-02 | 6.65E-02 |
| Black | Wnt signaling pathway | 0.014607258 | 8.33E-01 | 8.18E-01 | 4.16E-01 |
| Black | Oxidative phosphorylation | 0.024571538 | 1.00E+00 | 9.55E-01 | 4.42E-01 |
| Black | Dorso-ventral axis formation | 0.030997344 | 1.00E+00 | 9.55E-01 | 4.42E-01 |
| Black | Basal transcription factors | 0.045256449 | 1.00E+00 | 9.55E-01 | 4.68E-01 |
| Black | Inositol phosphate metabolism | 0.049264496 | 1.00E+00 | 9.55E-01 | 4.68E-01 |
| Black | Insulin signaling pathway | 0.104594871 | 1.00E+00 | 9.55E-01 | 6.62E-01 |
| Black | Neuroactive ligand-receptor interaction | 0.104594871 | 1.00E+00 | 9.55E-01 | 6.62E-01 |
| Black | Tight junction | 0.104594871 | 1.00E+00 | 9.55E-01 | 6.62E-01 |
| Black | Valine, leucine and isoleucine biosynthesis | 0.12908778 | 1.00E+00 | 9.55E-01 | 6.69E-01 |
| Black | Parkinson's disease | 0.12908778 | 1.00E+00 | 9.55E-01 | 6.69E-01 |
| Black | Histidine metabolism | 0.174882447 | 1.00E+00 | 9.55E-01 | 6.89E-01 |
| Black | Phosphatidylinositol signaling system | 0.193203641 | 1.00E+00 | 9.55E-01 | 6.89E-01 |
| Black | Chondroitin sulfate biosynthesis | 0.225129628 | 1.00E+00 | 9.55E-01 | 6.89E-01 |
| Black | Hedgehog signaling pathway | 0.225129628 | 1.00E+00 | 9.55E-01 | 6.89E-01 |
| Black | Cell cycle | 0.235430677 | 1.00E+00 | 9.55E-01 | 6.89E-01 |
| Black | Fatty acid biosynthesis | 0.241705739 | 1.00E+00 | 9.55E-01 | 6.89E-01 |
| Black | Ethylbenzene degradation | 0.241705739 | 1.00E+00 | 9.55E-01 | 6.89E-01 |
| Black | C21-Steroid hormone metabolism | 0.241705739 | 1.00E+00 | 9.55E-01 | 6.89E-01 |
| Black | Aminoacyl-tRNA biosynthesis | 0.241705739 | 1.00E+00 | 9.55E-01 | 6.89E-01 |
| Black | Glycosphingolipid metabolism | 0.276295964 | 1.00E+00 | 9.55E-01 | 7.50E-01 |
| Black | Tyrosine metabolism | 0.327321979 | 1.00E+00 | 9.55E-01 | 8.42E-01 |
| Black | 1- and 2-Methylnaphthalene degradation | 0.339930384 | 1.00E+00 | 9.55E-01 | 8.42E-01 |
| Black | Nicotinate and nicotinamide metabolism | 0.377400177 | 1.00E+00 | 9.55E-01 | 8.60E-01 |
| Black | ABC transporters - Organism-specific | 0.377400177 | 1.00E+00 | 9.55E-01 | 8.60E-01 |
| Black | Regulation of actin cytoskeleton | 0.41465972 | 1.00E+00 | 9.55E-01 | 8.66E-01 |
| Black | Androgen and estrogen metabolism | 0.425579556 | 1.00E+00 | 9.55E-01 | 8.66E-01 |
| Black | Alkaloid biosynthesis II | 0.425579556 | 1.00E+00 | 9.55E-01 | 8.66E-01 |
| Black | Gap junction | 0.453496309 | 1.00E+00 | 9.55E-01 | 8.66E-01 |
| Black | Benzoate degradation via CoA ligation | 0.472474025 | 1.00E+00 | 9.55E-01 | 8.66E-01 |
| Black | Limonene and pinene degradation | 0.500244258 | 1.00E+00 | 9.55E-01 | 8.66E-01 |
| Black | Glycosylphosphatidylinositol(GPI)-anchor biosynthesis | 0.500244258 | 1.00E+00 | 9.55E-01 | 8.66E-01 |
| Black | Cytokine-cytokine receptor interaction | 0.540943126 | 1.00E+00 | 9.55E-01 | 8.66E-01 |
| Black | TGF-beta signaling pathway | 0.558523986 | 1.00E+00 | 9.55E-01 | 8.66E-01 |
| Black | Focal adhesion | 0.560752163 | 1.00E+00 | 9.55E-01 | 8.66E-01 |
| Black | Phenylalanine metabolism | 0.565316621 | 1.00E+00 | 9.55E-01 | 8.66E-01 |
| Black | Proteasome | 0.565316621 | 1.00E+00 | 9.55E-01 | 8.66E-01 |
| Black | Calcium signaling pathway | 0.589176 | 1.00E+00 | 9.55E-01 | 8.66E-01 |
| Black | Glutamate metabolism | 0.622014453 | 1.00E+00 | 9.55E-01 | 8.66E-01 |
| Black | Propanoate metabolism | 0.622014453 | 1.00E+00 | 9.55E-01 | 8.66E-01 |
| Black | Adherens junction | 0.634323269 | 1.00E+00 | 9.55E-01 | 8.66E-01 |
| Black | Alzheimer's disease | 0.671402808 | 1.00E+00 | 9.55E-01 | 8.66E-01 |
| Black | Valine, leucine and isoleucine degradation | 0.671402808 | 1.00E+00 | 9.55E-01 | 8.66E-01 |
| Black | MAPK signaling pathway | 0.675829423 | 1.00E+00 | 9.55E-01 | 8.66E-01 |
| Black | Jak-STAT signaling pathway | 0.701851851 | 1.00E+00 | 9.55E-01 | 8.66E-01 |
| Black | Butanoate metabolism | 0.714412912 | 1.00E+00 | 9.55E-01 | 8.66E-01 |
| Black | Notch signaling pathway | 0.714412912 | 1.00E+00 | 9.55E-01 | 8.66E-01 |
| Black | Glutathione metabolism | 0.751858771 | 1.00E+00 | 9.55E-01 | 8.75E-01 |
| Black | Apoptosis | 0.751858771 | 1.00E+00 | 9.55E-01 | 8.75E-01 |
| Black | Pyruvate metabolism | 0.812813364 | 1.00E+00 | 9.55E-01 | 9.08E-01 |
| Black | Lysine degradation | 0.812813364 | 1.00E+00 | 9.55E-01 | 9.08E-01 |
| Black | Purine metabolism | 0.85730963 | 1.00E+00 | 9.55E-01 | 9.26E-01 |
| Black | Hematopoietic cell lineage | 0.872417154 | 1.00E+00 | 9.55E-01 | 9.26E-01 |
| Black | Ribosome | 0.877603176 | 1.00E+00 | 9.55E-01 | 9.26E-01 |
| Black | Glycerophospholipid metabolism | 0.920161458 | 1.00E+00 | 9.55E-01 | 9.47E-01 |
| Black | Tryptophan metabolism | 0.930797143 | 1.00E+00 | 9.55E-01 | 9.47E-01 |
| Black | Complement and coagulation cascades | 0.95500712 | 1.00E+00 | 9.55E-01 | 9.55E-01 |
| Blue | ECM-receptor interaction | 2.26E-09 | 1.81E-07 | 1.81E-07 | 1.81E-07 |
| Blue | Complement and coagulation cascades | 1.01E-06 | 8.10E-05 | 8.00E-05 | 4.05E-05 |
| Blue | Focal adhesion | 0.000630915 | 5.05E-02 | 4.92E-02 | 1.68E-02 |
| Blue | Fatty acid metabolism | 0.020744284 | 1.00E+00 | 9.98E-01 | 3.93E-01 |
| Blue | Tryptophan metabolism | 0.024558455 | 1.00E+00 | 9.98E-01 | 3.93E-01 |
| Blue | Novobiocin biosynthesis | 0.041670129 | 1.00E+00 | 9.98E-01 | 4.17E-01 |
| Blue | Alkaloid biosynthesis I | 0.041670129 | 1.00E+00 | 9.98E-01 | 4.17E-01 |
| Blue | beta-Alanine metabolism | 0.041670129 | 1.00E+00 | 9.98E-01 | 4.17E-01 |
| Blue | gamma-Hexachlorocyclohexane degradation | 0.05950632 | 1.00E+00 | 9.98E-01 | 5.29E-01 |
| Blue | Phenylalanine, tyrosine and tryptophan biosynthesis | 0.108169609 | 1.00E+00 | 9.98E-01 | 7.87E-01 |
| Blue | Pentose and glucuronate interconversions | 0.108169609 | 1.00E+00 | 9.98E-01 | 7.87E-01 |
| Blue | Glutathione metabolism | 0.12767871 | 1.00E+00 | 9.98E-01 | 8.19E-01 |
| Blue | TGF-beta signaling pathway | 0.137797399 | 1.00E+00 | 9.98E-01 | 8.19E-01 |
| Blue | Inositol phosphate metabolism | 0.155241863 | 1.00E+00 | 9.98E-01 | 8.19E-01 |
| Blue | Hedgehog signaling pathway | 0.155241863 | 1.00E+00 | 9.98E-01 | 8.19E-01 |
| Blue | Gap junction | 0.173700946 | 1.00E+00 | 9.98E-01 | 8.19E-01 |
| Blue | Methionine metabolism | 0.187727378 | 1.00E+00 | 9.98E-01 | 8.19E-01 |
| Blue | Retinol metabolism | 0.204819277 | 1.00E+00 | 9.98E-01 | 8.19E-01 |
| Blue | Caprolactam degradation | 0.204819277 | 1.00E+00 | 9.98E-01 | 8.19E-01 |
| Blue | Vitamin B6 metabolism | 0.204819277 | 1.00E+00 | 9.98E-01 | 8.19E-01 |
| Blue | Benzoate degradation via CoA ligation | 0.216610865 | 1.00E+00 | 9.98E-01 | 8.25E-01 |
| Blue | Cysteine metabolism | 0.272294851 | 1.00E+00 | 9.98E-01 | 9.13E-01 |
| Blue | Notch signaling pathway | 0.273766811 | 1.00E+00 | 9.98E-01 | 9.13E-01 |
| Blue | Tyrosine metabolism | 0.273766811 | 1.00E+00 | 9.98E-01 | 9.13E-01 |
| Blue | Nicotinate and nicotinamide metabolism | 0.336375997 | 1.00E+00 | 9.98E-01 | 9.24E-01 |
| Blue | Nitrogen metabolism | 0.356531982 | 1.00E+00 | 9.98E-01 | 9.24E-01 |
| Blue | Alanine and aspartate metabolism | 0.356531982 | 1.00E+00 | 9.98E-01 | 9.24E-01 |
| Blue | Phenylalanine metabolism | 0.356531982 | 1.00E+00 | 9.98E-01 | 9.24E-01 |
| Blue | Tetrachloroethene degradation | 0.367968425 | 1.00E+00 | 9.98E-01 | 9.24E-01 |
| Blue | Pantothenate and CoA biosynthesis | 0.367968425 | 1.00E+00 | 9.98E-01 | 9.24E-01 |
| Blue | Sulfur metabolism | 0.367968425 | 1.00E+00 | 9.98E-01 | 9.24E-01 |
| Blue | Pyrimidine metabolism | 0.369521824 | 1.00E+00 | 9.98E-01 | 9.24E-01 |
| Blue | Cytokine-cytokine receptor interaction | 0.398512223 | 1.00E+00 | 9.98E-01 | 9.66E-01 |
| Blue | Chondroitin sulfate biosynthesis | 0.437048029 | 1.00E+00 | 9.98E-01 | 9.93E-01 |
| Blue | Selenoamino acid metabolism | 0.437048029 | 1.00E+00 | 9.98E-01 | 9.93E-01 |
| Blue | Hematopoietic cell lineage | 0.447586366 | 1.00E+00 | 9.98E-01 | 9.93E-01 |
| Blue | Phosphatidylinositol signaling system | 0.459447677 | 1.00E+00 | 9.98E-01 | 9.93E-01 |
| Blue | Fatty acid elongation in mitochondria | 0.497867834 | 1.00E+00 | 9.98E-01 | 9.98E-01 |
| Blue | One carbon pool by folate | 0.497867834 | 1.00E+00 | 9.98E-01 | 9.98E-01 |
| Blue | Arginine and proline metabolism | 0.511836308 | 1.00E+00 | 9.98E-01 | 9.98E-01 |
| Blue | Valine, leucine and isoleucine degradation | 0.511836308 | 1.00E+00 | 9.98E-01 | 9.98E-01 |
| Blue | MAPK signaling pathway | 0.534764522 | 1.00E+00 | 9.98E-01 | 9.98E-01 |
| Blue | Alkaloid biosynthesis II | 0.601247986 | 1.00E+00 | 9.98E-01 | 9.98E-01 |
| Blue | Streptomycin biosynthesis | 0.601247986 | 1.00E+00 | 9.98E-01 | 9.98E-01 |
| Blue | Blood group glycolipid biosynthesis - neo-lactoseries | 0.601247986 | 1.00E+00 | 9.98E-01 | 9.98E-01 |
| Blue | Blood group glycolipid biosynthesis - lactoseries | 0.601247986 | 1.00E+00 | 9.98E-01 | 9.98E-01 |
| Blue | Apoptosis | 0.640726207 | 1.00E+00 | 9.98E-01 | 9.98E-01 |
| Blue | ABC transporters - Organism-specific | 0.640726207 | 1.00E+00 | 9.98E-01 | 9.98E-01 |
| Blue | Tight junction | 0.644349491 | 1.00E+00 | 9.98E-01 | 9.98E-01 |
| Blue | Neuroactive ligand-receptor interaction | 0.644349491 | 1.00E+00 | 9.98E-01 | 9.98E-01 |
| Blue | Nucleotide sugars metabolism | 0.683486269 | 1.00E+00 | 9.98E-01 | 9.98E-01 |
| Blue | Glycosylphosphatidylinositol(GPI)-anchor biosynthesis | 0.683486269 | 1.00E+00 | 9.98E-01 | 9.98E-01 |
| Blue | Limonene and pinene degradation | 0.683486269 | 1.00E+00 | 9.98E-01 | 9.98E-01 |
| Blue | Calcium signaling pathway | 0.727907193 | 1.00E+00 | 9.98E-01 | 9.98E-01 |
| Blue | Pyruvate metabolism | 0.741511975 | 1.00E+00 | 9.98E-01 | 9.98E-01 |
| Blue | Histidine metabolism | 0.748877127 | 1.00E+00 | 9.98E-01 | 9.98E-01 |
| Blue | Glycine, serine and threonine metabolism | 0.748877127 | 1.00E+00 | 9.98E-01 | 9.98E-01 |
| Blue | Dorso-ventral axis formation | 0.748877127 | 1.00E+00 | 9.98E-01 | 9.98E-01 |
| Blue | Propanoate metabolism | 0.800848643 | 1.00E+00 | 9.98E-01 | 9.98E-01 |
| Blue | O-Glycan biosynthesis | 0.800848643 | 1.00E+00 | 9.98E-01 | 9.98E-01 |
| Blue | Glutamate metabolism | 0.800848643 | 1.00E+00 | 9.98E-01 | 9.98E-01 |
| Blue | Carbon fixation | 0.800848643 | 1.00E+00 | 9.98E-01 | 9.98E-01 |
| Blue | Glycosphingolipid metabolism | 0.84213612 | 1.00E+00 | 9.98E-01 | 9.98E-01 |
| Blue | Alzheimer's disease | 0.84213612 | 1.00E+00 | 9.98E-01 | 9.98E-01 |
| Blue | Adherens junction | 0.872702582 | 1.00E+00 | 9.98E-01 | 9.98E-01 |
| Blue | Butanoate metabolism | 0.874921114 | 1.00E+00 | 9.98E-01 | 9.98E-01 |
| Blue | Aminosugars metabolism | 0.874921114 | 1.00E+00 | 9.98E-01 | 9.98E-01 |
| Blue | Starch and sucrose metabolism | 0.90094277 | 1.00E+00 | 9.98E-01 | 9.98E-01 |
| Blue | Purine metabolism | 0.915908843 | 1.00E+00 | 9.98E-01 | 9.98E-01 |
| Blue | Galactose metabolism | 0.921586922 | 1.00E+00 | 9.98E-01 | 9.98E-01 |
| Blue | Fructose and mannose metabolism | 0.937957371 | 1.00E+00 | 9.98E-01 | 9.98E-01 |
| Blue | Lysine degradation | 0.937957371 | 1.00E+00 | 9.98E-01 | 9.98E-01 |
| Blue | Glycerolipid metabolism | 0.937957371 | 1.00E+00 | 9.98E-01 | 9.98E-01 |
| Blue | Jak-STAT signaling pathway | 0.940146837 | 1.00E+00 | 9.98E-01 | 9.98E-01 |
| Blue | Regulation of actin cytoskeleton | 0.940363764 | 1.00E+00 | 9.98E-01 | 9.98E-01 |
| Blue | Toll-like receptor signaling pathway | 0.975796705 | 1.00E+00 | 9.98E-01 | 9.98E-01 |
| Blue | Wnt signaling pathway | 0.98089439 | 1.00E+00 | 9.98E-01 | 9.98E-01 |
| Blue | Insulin signaling pathway | 0.982019292 | 1.00E+00 | 9.98E-01 | 9.98E-01 |
| Blue | Glycolysis / Gluconeogenesis | 0.988111792 | 1.00E+00 | 9.98E-01 | 9.98E-01 |
| Blue | Cell cycle | 0.99777469 | 1.00E+00 | 9.98E-01 | 9.98E-01 |
| Brown | Biosynthesis of steroids | 2.25E-06 | 1.80E-04 | 1.77E-04 | 8.98E-05 |
| Brown | Biosynthesis of steroids | 2.25E-06 | 1.80E-04 | 1.77E-04 | 8.98E-05 |
| Brown | Glycolysis / Gluconeogenesis | 1.52E-05 | 1.21E-03 | 1.18E-03 | 4.05E-04 |
| Brown | Pyruvate metabolism | 0.001381015 | 1.10E-01 | 1.06E-01 | 2.76E-02 |
| Brown | Terpenoid biosynthesis | 0.001757611 | 1.41E-01 | 1.34E-01 | 2.81E-02 |
| Brown | Oxidative phosphorylation | 0.003665283 | 2.93E-01 | 2.75E-01 | 4.89E-02 |
| Brown | Nucleotide sugars metabolism | 0.014618508 | 1.00E+00 | 9.85E-01 | 1.28E-01 |
| Brown | Fatty acid biosynthesis | 0.01474865 | 1.00E+00 | 9.85E-01 | 1.28E-01 |
| Brown | Glycerophospholipid metabolism | 0.015134217 | 1.00E+00 | 9.85E-01 | 1.28E-01 |
| Brown | Butanoate metabolism | 0.016050466 | 1.00E+00 | 9.85E-01 | 1.28E-01 |
| Brown | Galactose metabolism | 0.034711948 | 1.00E+00 | 9.85E-01 | 2.52E-01 |
| Brown | Fatty acid elongation in mitochondria | 0.040730727 | 1.00E+00 | 9.85E-01 | 2.52E-01 |
| Brown | Propanoate metabolism | 0.042642137 | 1.00E+00 | 9.85E-01 | 2.52E-01 |
| Brown | Fructose and mannose metabolism | 0.047340344 | 1.00E+00 | 9.85E-01 | 2.52E-01 |
| Brown | Benzoate degradation via CoA ligation | 0.047340344 | 1.00E+00 | 9.85E-01 | 2.52E-01 |
| Brown | Pentose phosphate pathway | 0.075051343 | 1.00E+00 | 9.85E-01 | 3.53E-01 |
| Brown | Citrate cycle (TCA cycle) | 0.075051343 | 1.00E+00 | 9.85E-01 | 3.53E-01 |
| Brown | Fatty acid metabolism | 0.085479817 | 1.00E+00 | 9.85E-01 | 3.80E-01 |
| Brown | Starch and sucrose metabolism | 0.111697894 | 1.00E+00 | 9.85E-01 | 4.25E-01 |
| Brown | Inositol metabolism | 0.122203098 | 1.00E+00 | 9.85E-01 | 4.25E-01 |
| Brown | Amyotrophic lateral sclerosis (ALS) | 0.122203098 | 1.00E+00 | 9.85E-01 | 4.25E-01 |
| Brown | Ascorbate and aldarate metabolism | 0.122203098 | 1.00E+00 | 9.85E-01 | 4.25E-01 |
| Brown | Peptidoglycan biosynthesis | 0.122203098 | 1.00E+00 | 9.85E-01 | 4.25E-01 |
| Brown | Lysine degradation | 0.171643384 | 1.00E+00 | 9.85E-01 | 5.72E-01 |
| Brown | Tryptophan metabolism | 0.192580812 | 1.00E+00 | 9.85E-01 | 6.12E-01 |
| Brown | Carbon fixation | 0.206478071 | 1.00E+00 | 9.85E-01 | 6.12E-01 |
| Brown | Ethylbenzene degradation | 0.229657546 | 1.00E+00 | 9.85E-01 | 6.12E-01 |
| Brown | Synthesis and degradation of ketone bodies | 0.229657546 | 1.00E+00 | 9.85E-01 | 6.12E-01 |
| Brown | Tetrachloroethene degradation | 0.229657546 | 1.00E+00 | 9.85E-01 | 6.12E-01 |
| Brown | Methane metabolism | 0.229657546 | 1.00E+00 | 9.85E-01 | 6.12E-01 |
| Brown | Valine, leucine and isoleucine degradation | 0.254525194 | 1.00E+00 | 9.85E-01 | 6.57E-01 |
| Brown | Aminosugars metabolism | 0.302823873 | 1.00E+00 | 9.85E-01 | 6.65E-01 |
| Brown | 1- and 2-Methylnaphthalene degradation | 0.324120956 | 1.00E+00 | 9.85E-01 | 6.65E-01 |
| Brown | Folate biosynthesis | 0.324120956 | 1.00E+00 | 9.85E-01 | 6.65E-01 |
| Brown | Phenylalanine, tyrosine and tryptophan biosynthesis | 0.324120956 | 1.00E+00 | 9.85E-01 | 6.65E-01 |
| Brown | Reductive carboxylate cycle (CO2 fixation) | 0.324120956 | 1.00E+00 | 9.85E-01 | 6.65E-01 |
| Brown | Bisphenol A degradation | 0.324120956 | 1.00E+00 | 9.85E-01 | 6.65E-01 |
| Brown | Porphyrin and chlorophyll metabolism | 0.324120956 | 1.00E+00 | 9.85E-01 | 6.65E-01 |
| Brown | Aminophosphonate metabolism | 0.324120956 | 1.00E+00 | 9.85E-01 | 6.65E-01 |
| Brown | Glutathione metabolism | 0.350605368 | 1.00E+00 | 9.85E-01 | 7.01E-01 |
| Brown | Glyoxylate and dicarboxylate metabolism | 0.40714416 | 1.00E+00 | 9.85E-01 | 7.24E-01 |
| Brown | Streptomycin biosynthesis | 0.40714416 | 1.00E+00 | 9.85E-01 | 7.24E-01 |
| Brown | Bile acid biosynthesis | 0.40714416 | 1.00E+00 | 9.85E-01 | 7.24E-01 |
| Brown | Neurodegenerative Disorders | 0.40714416 | 1.00E+00 | 9.85E-01 | 7.24E-01 |
| Brown | Alkaloid biosynthesis II | 0.40714416 | 1.00E+00 | 9.85E-01 | 7.24E-01 |
| Brown | Jak-STAT signaling pathway | 0.424690998 | 1.00E+00 | 9.85E-01 | 7.39E-01 |
| Brown | Arachidonic acid metabolism | 0.480095226 | 1.00E+00 | 9.85E-01 | 7.77E-01 |
| Brown | Limonene and pinene degradation | 0.480095226 | 1.00E+00 | 9.85E-01 | 7.77E-01 |
| Brown | Urea cycle and metabolism of amino groups | 0.54418071 | 1.00E+00 | 9.85E-01 | 7.77E-01 |
| Brown | Glycine, serine and threonine metabolism | 0.54418071 | 1.00E+00 | 9.85E-01 | 7.77E-01 |
| Brown | Huntington's disease | 0.54418071 | 1.00E+00 | 9.85E-01 | 7.77E-01 |
| Brown | Alanine and aspartate metabolism | 0.54418071 | 1.00E+00 | 9.85E-01 | 7.77E-01 |
| Brown | Phenylalanine metabolism | 0.54418071 | 1.00E+00 | 9.85E-01 | 7.77E-01 |
| Brown | Histidine metabolism | 0.54418071 | 1.00E+00 | 9.85E-01 | 7.77E-01 |
| Brown | Nitrogen metabolism | 0.54418071 | 1.00E+00 | 9.85E-01 | 7.77E-01 |
| Brown | Dentatorubropallidoluysian atrophy (DRPLA) | 0.54418071 | 1.00E+00 | 9.85E-01 | 7.77E-01 |
| Brown | Ribosome | 0.565782986 | 1.00E+00 | 9.85E-01 | 7.94E-01 |
| Brown | Glutamate metabolism | 0.600464483 | 1.00E+00 | 9.85E-01 | 8.28E-01 |
| Brown | Insulin signaling pathway | 0.63693492 | 1.00E+00 | 9.85E-01 | 8.39E-01 |
| Brown | Alzheimer's disease | 0.649884382 | 1.00E+00 | 9.85E-01 | 8.39E-01 |
| Brown | gamma-Hexachlorocyclohexane degradation | 0.649884382 | 1.00E+00 | 9.85E-01 | 8.39E-01 |
| Brown | Arginine and proline metabolism | 0.649884382 | 1.00E+00 | 9.85E-01 | 8.39E-01 |
| Brown | Tyrosine metabolism | 0.693266945 | 1.00E+00 | 9.85E-01 | 8.80E-01 |
| Brown | Nicotinate and nicotinamide metabolism | 0.731340454 | 1.00E+00 | 9.85E-01 | 9.00E-01 |
| Brown | Apoptosis | 0.731340454 | 1.00E+00 | 9.85E-01 | 9.00E-01 |
| Brown | Phosphatidylinositol signaling system | 0.794049968 | 1.00E+00 | 9.85E-01 | 9.48E-01 |
| Brown | Glycerolipid metabolism | 0.794049968 | 1.00E+00 | 9.85E-01 | 9.48E-01 |
| Brown | Purine metabolism | 0.833917156 | 1.00E+00 | 9.85E-01 | 9.68E-01 |
| Brown | Tight junction | 0.850332342 | 1.00E+00 | 9.85E-01 | 9.68E-01 |
| Brown | Hematopoietic cell lineage | 0.850332342 | 1.00E+00 | 9.85E-01 | 9.68E-01 |
| Brown | Adherens junction | 0.879336928 | 1.00E+00 | 9.85E-01 | 9.68E-01 |
| Brown | Regulation of actin cytoskeleton | 0.890600124 | 1.00E+00 | 9.85E-01 | 9.68E-01 |
| Brown | Wnt signaling pathway | 0.894499898 | 1.00E+00 | 9.85E-01 | 9.68E-01 |
| Brown | Cytokine-cytokine receptor interaction | 0.912305447 | 1.00E+00 | 9.85E-01 | 9.68E-01 |
| Brown | MAPK signaling pathway | 0.918066143 | 1.00E+00 | 9.85E-01 | 9.68E-01 |
| Brown | Gap junction | 0.919410697 | 1.00E+00 | 9.85E-01 | 9.68E-01 |
| Brown | Complement and coagulation cascades | 0.94629971 | 1.00E+00 | 9.85E-01 | 9.78E-01 |
| Brown | Calcium signaling pathway | 0.953120319 | 1.00E+00 | 9.85E-01 | 9.78E-01 |
| Brown | Neuroactive ligand-receptor interaction | 0.968859368 | 1.00E+00 | 9.85E-01 | 9.81E-01 |
| Brown | Focal adhesion | 0.98464065 | 1.00E+00 | 9.85E-01 | 9.85E-01 |
| Cyan | Regulation of actin cytoskeleton | 0.008785603 | 1.14E-01 | 1.14E-01 | 7.79E-02 |
| Cyan | Calcium signaling pathway | 0.011981047 | 1.56E-01 | 1.44E-01 | 7.79E-02 |
| Cyan | Atrazine degradation | 0.022375215 | 2.91E-01 | 2.46E-01 | 9.70E-02 |
| Cyan | Adherens junction | 0.046492517 | 6.04E-01 | 4.39E-01 | 1.51E-01 |
| Cyan | Glycolysis / Gluconeogenesis | 0.06378061 | 8.29E-01 | 4.39E-01 | 1.66E-01 |
| Cyan | Cysteine metabolism | 0.107333912 | 1.00E+00 | 4.39E-01 | 2.06E-01 |
| Cyan | Tight junction | 0.110956554 | 1.00E+00 | 4.39E-01 | 2.06E-01 |
| Cyan | Propanoate metabolism | 0.147207409 | 1.00E+00 | 4.39E-01 | 2.39E-01 |
| Cyan | Pyruvate metabolism | 0.239810263 | 1.00E+00 | 4.39E-01 | 3.15E-01 |
| Cyan | Oxidative phosphorylation | 0.274179593 | 1.00E+00 | 4.39E-01 | 3.15E-01 |
| Cyan | Focal adhesion | 0.275023407 | 1.00E+00 | 4.39E-01 | 3.15E-01 |
| Cyan | Ribosome | 0.290820978 | 1.00E+00 | 4.39E-01 | 3.15E-01 |
| Cyan | Purine metabolism | 0.438923773 | 1.00E+00 | 4.39E-01 | 4.39E-01 |
| Green | Toll-like receptor signaling pathway | 1.59E-08 | 1.05E-06 | 1.05E-06 | 1.05E-06 |
| Green | Cytokine-cytokine receptor interaction | 2.87E-07 | 1.90E-05 | 1.87E-05 | 9.49E-06 |
| Green | Hematopoietic cell lineage | 6.57E-05 | 4.33E-03 | 4.20E-03 | 1.44E-03 |
| Green | Jak-STAT signaling pathway | 0.002245403 | 1.48E-01 | 1.41E-01 | 3.70E-02 |
| Green | MAPK signaling pathway | 0.01759658 | 1.00E+00 | 9.72E-01 | 1.95E-01 |
| Green | O-Glycan biosynthesis | 0.017736857 | 1.00E+00 | 9.72E-01 | 1.95E-01 |
| Green | Globoside metabolism | 0.035992299 | 1.00E+00 | 9.72E-01 | 3.39E-01 |
| Green | Urea cycle and metabolism of amino groups | 0.06325692 | 1.00E+00 | 9.72E-01 | 4.97E-01 |
| Green | One carbon pool by folate | 0.075273759 | 1.00E+00 | 9.72E-01 | 4.97E-01 |
| Green | High-mannose type N-glycan biosynthesis | 0.075273759 | 1.00E+00 | 9.72E-01 | 4.97E-01 |
| Green | Regulation of actin cytoskeleton | 0.116700654 | 1.00E+00 | 9.72E-01 | 5.53E-01 |
| Green | Glycerolipid metabolism | 0.127328321 | 1.00E+00 | 9.72E-01 | 5.53E-01 |
| Green | Fructose and mannose metabolism | 0.127328321 | 1.00E+00 | 9.72E-01 | 5.53E-01 |
| Green | Blood group glycolipid biosynthesis - lactoseries | 0.134144934 | 1.00E+00 | 9.72E-01 | 5.53E-01 |
| Green | Blood group glycolipid biosynthesis - neo-lactoseries | 0.134144934 | 1.00E+00 | 9.72E-01 | 5.53E-01 |
| Green | Ganglioside biosynthesis | 0.134144934 | 1.00E+00 | 9.72E-01 | 5.53E-01 |
| Green | Glycosylphosphatidylinositol(GPI)-anchor biosynthesis | 0.199580024 | 1.00E+00 | 9.72E-01 | 7.32E-01 |
| Green | Arachidonic acid metabolism | 0.199580024 | 1.00E+00 | 9.72E-01 | 7.32E-01 |
| Green | Apoptosis | 0.229212014 | 1.00E+00 | 9.72E-01 | 7.96E-01 |
| Green | N-Glycan biosynthesis | 0.267741577 | 1.00E+00 | 9.72E-01 | 8.84E-01 |
| Green | N-Glycan degradation | 0.309140008 | 1.00E+00 | 9.72E-01 | 8.87E-01 |
| Green | Synthesis and degradation of ketone bodies | 0.309140008 | 1.00E+00 | 9.72E-01 | 8.87E-01 |
| Green | Glycosaminoglycan degradation | 0.309140008 | 1.00E+00 | 9.72E-01 | 8.87E-01 |
| Green | Glycerophospholipid metabolism | 0.360490321 | 1.00E+00 | 9.72E-01 | 9.61E-01 |
| Green | Glycosphingolipid metabolism | 0.402078383 | 1.00E+00 | 9.72E-01 | 9.61E-01 |
| Green | Arginine and proline metabolism | 0.402078383 | 1.00E+00 | 9.72E-01 | 9.61E-01 |
| Green | TGF-beta signaling pathway | 0.429390674 | 1.00E+00 | 9.72E-01 | 9.61E-01 |
| Green | Aminosugars metabolism | 0.465058996 | 1.00E+00 | 9.72E-01 | 9.61E-01 |
| Green | Neurodegenerative Disorders | 0.523382533 | 1.00E+00 | 9.72E-01 | 9.61E-01 |
| Green | Pentose phosphate pathway | 0.523382533 | 1.00E+00 | 9.72E-01 | 9.61E-01 |
| Green | Streptomycin biosynthesis | 0.523382533 | 1.00E+00 | 9.72E-01 | 9.61E-01 |
| Green | Methionine metabolism | 0.523382533 | 1.00E+00 | 9.72E-01 | 9.61E-01 |
| Green | Glyoxylate and dicarboxylate metabolism | 0.523382533 | 1.00E+00 | 9.72E-01 | 9.61E-01 |
| Green | Bile acid biosynthesis | 0.523382533 | 1.00E+00 | 9.72E-01 | 9.61E-01 |
| Green | Adherens junction | 0.523939629 | 1.00E+00 | 9.72E-01 | 9.61E-01 |
| Green | ABC transporters - Organism-specific | 0.524020741 | 1.00E+00 | 9.72E-01 | 9.61E-01 |
| Green | Galactose metabolism | 0.578519339 | 1.00E+00 | 9.72E-01 | 9.61E-01 |
| Green | Limonene and pinene degradation | 0.60433316 | 1.00E+00 | 9.72E-01 | 9.61E-01 |
| Green | Pyruvate metabolism | 0.628371213 | 1.00E+00 | 9.72E-01 | 9.61E-01 |
| Green | Glycolysis / Gluconeogenesis | 0.648271963 | 1.00E+00 | 9.72E-01 | 9.61E-01 |
| Green | Insulin signaling pathway | 0.665859038 | 1.00E+00 | 9.72E-01 | 9.61E-01 |
| Green | Glycine, serine and threonine metabolism | 0.671651476 | 1.00E+00 | 9.72E-01 | 9.61E-01 |
| Green | Huntington's disease | 0.671651476 | 1.00E+00 | 9.72E-01 | 9.61E-01 |
| Green | Nitrogen metabolism | 0.671651476 | 1.00E+00 | 9.72E-01 | 9.61E-01 |
| Green | Dentatorubropallidoluysian atrophy (DRPLA) | 0.671651476 | 1.00E+00 | 9.72E-01 | 9.61E-01 |
| Green | Carbon fixation | 0.727613485 | 1.00E+00 | 9.72E-01 | 9.61E-01 |
| Green | Chondroitin sulfate biosynthesis | 0.727613485 | 1.00E+00 | 9.72E-01 | 9.61E-01 |
| Green | Complement and coagulation cascades | 0.748102288 | 1.00E+00 | 9.72E-01 | 9.61E-01 |
| Green | Pyrimidine metabolism | 0.750685779 | 1.00E+00 | 9.72E-01 | 9.61E-01 |
| Green | Valine, leucine and isoleucine degradation | 0.7741185 | 1.00E+00 | 9.72E-01 | 9.61E-01 |
| Green | Alzheimer's disease | 0.7741185 | 1.00E+00 | 9.72E-01 | 9.61E-01 |
| Green | Calcium signaling pathway | 0.775975775 | 1.00E+00 | 9.72E-01 | 9.61E-01 |
| Green | Wnt signaling pathway | 0.811759133 | 1.00E+00 | 9.72E-01 | 9.61E-01 |
| Green | Butanoate metabolism | 0.812750938 | 1.00E+00 | 9.72E-01 | 9.61E-01 |
| Green | Starch and sucrose metabolism | 0.844832071 | 1.00E+00 | 9.72E-01 | 9.61E-01 |
| Green | Glutathione metabolism | 0.844832071 | 1.00E+00 | 9.72E-01 | 9.61E-01 |
| Green | Nicotinate and nicotinamide metabolism | 0.844832071 | 1.00E+00 | 9.72E-01 | 9.61E-01 |
| Green | Tight junction | 0.8448835 | 1.00E+00 | 9.72E-01 | 9.61E-01 |
| Green | Phosphatidylinositol signaling system | 0.893562629 | 1.00E+00 | 9.72E-01 | 9.72E-01 |
| Green | Benzoate degradation via CoA ligation | 0.893562629 | 1.00E+00 | 9.72E-01 | 9.72E-01 |
| Green | Focal adhesion | 0.915887377 | 1.00E+00 | 9.72E-01 | 9.72E-01 |
| Green | Purine metabolism | 0.943863371 | 1.00E+00 | 9.72E-01 | 9.72E-01 |
| Green | Neuroactive ligand-receptor interaction | 0.952111693 | 1.00E+00 | 9.72E-01 | 9.72E-01 |
| Green | Cell cycle | 0.952111693 | 1.00E+00 | 9.72E-01 | 9.72E-01 |
| Green | ECM-receptor interaction | 0.971875079 | 1.00E+00 | 9.72E-01 | 9.72E-01 |
| Green | Gap junction | 0.971875079 | 1.00E+00 | 9.72E-01 | 9.72E-01 |
| GreenYellow | Cell cycle | 6.26E-18 | 8.77E-17 | 8.77E-17 | 8.77E-17 |
| GreenYellow | DNA polymerase | 0.004018289 | 5.63E-02 | 5.22E-02 | 2.81E-02 |
| GreenYellow | Pyrimidine metabolism | 0.015979849 | 2.24E-01 | 1.92E-01 | 7.46E-02 |
| GreenYellow | Calcium signaling pathway | 0.051009867 | 7.14E-01 | 5.61E-01 | 1.77E-01 |
| GreenYellow | Purine metabolism | 0.063052288 | 8.83E-01 | 6.31E-01 | 1.77E-01 |
| GreenYellow | Inositol phosphate metabolism | 0.237873253 | 1.00E+00 | 8.22E-01 | 5.23E-01 |
| GreenYellow | ABC transporters - Organism-specific | 0.322330365 | 1.00E+00 | 8.22E-01 | 5.23E-01 |
| GreenYellow | Nicotinate and nicotinamide metabolism | 0.322330365 | 1.00E+00 | 8.22E-01 | 5.23E-01 |
| GreenYellow | Phosphatidylinositol signaling system | 0.373588149 | 1.00E+00 | 8.22E-01 | 5.23E-01 |
| GreenYellow | Benzoate degradation via CoA ligation | 0.373588149 | 1.00E+00 | 8.22E-01 | 5.23E-01 |
| GreenYellow | TGF-beta signaling pathway | 0.42113228 | 1.00E+00 | 8.22E-01 | 5.36E-01 |
| GreenYellow | Gap junction | 0.525391318 | 1.00E+00 | 8.22E-01 | 5.66E-01 |
| GreenYellow | Tryptophan metabolism | 0.525391318 | 1.00E+00 | 8.22E-01 | 5.66E-01 |
| GreenYellow | MAPK signaling pathway | 0.821692477 | 1.00E+00 | 8.22E-01 | 8.22E-01 |
| Grey | Neuroactive ligand-receptor interaction | 0.010359281 | 1.24E-01 | 1.24E-01 | 1.14E-01 |
| Grey | 2,4-Dichlorobenzoate degradation | 0.018932874 | 2.27E-01 | 2.08E-01 | 1.14E-01 |
| Grey | C21-Steroid hormone metabolism | 0.03753932 | 4.50E-01 | 3.75E-01 | 1.50E-01 |
| Grey | Alkaloid biosynthesis II | 0.07379315 | 8.86E-01 | 5.74E-01 | 1.77E-01 |
| Grey | Androgen and estrogen metabolism | 0.07379315 | 8.86E-01 | 5.74E-01 | 1.77E-01 |
| Grey | Glycosphingolipid metabolism | 0.142602263 | 1.00E+00 | 5.74E-01 | 2.73E-01 |
| Grey | Butanoate metabolism | 0.159061906 | 1.00E+00 | 5.74E-01 | 2.73E-01 |
| Grey | Lysine degradation | 0.206732376 | 1.00E+00 | 5.74E-01 | 2.76E-01 |
| Grey | Fructose and mannose metabolism | 0.206732376 | 1.00E+00 | 5.74E-01 | 2.76E-01 |
| Grey | Ribosome | 0.251933417 | 1.00E+00 | 5.74E-01 | 3.02E-01 |
| Grey | Complement and coagulation cascades | 0.348440228 | 1.00E+00 | 5.74E-01 | 3.80E-01 |
| Grey | MAPK signaling pathway | 0.574068366 | 1.00E+00 | 5.74E-01 | 5.74E-01 |
| LightCyan | Arachidonic acid metabolism | 0.003163858 | 5.06E-02 | 5.06E-02 | 3.76E-02 |
| LightCyan | Dentatorubropallidoluysian atrophy (DRPLA) | 0.004696612 | 7.51E-02 | 7.04E-02 | 3.76E-02 |
| LightCyan | Neuroactive ligand-receptor interaction | 0.010359281 | 1.66E-01 | 1.45E-01 | 5.18E-02 |
| LightCyan | Taurine and hypotaurine metabolism | 0.018932874 | 3.03E-01 | 2.14E-01 | 5.18E-02 |
| LightCyan | Cyanoamino acid metabolism | 0.018932874 | 3.03E-01 | 2.14E-01 | 5.18E-02 |
| LightCyan | Glycerolipid metabolism | 0.019413366 | 3.11E-01 | 2.14E-01 | 5.18E-02 |
| LightCyan | Bile acid biosynthesis | 0.07379315 | 1.00E+00 | 5.74E-01 | 1.65E-01 |
| LightCyan | Insulin signaling pathway | 0.082639956 | 1.00E+00 | 5.74E-01 | 1.65E-01 |
| LightCyan | Selenoamino acid metabolism | 0.125850265 | 1.00E+00 | 5.74E-01 | 2.24E-01 |
| LightCyan | Regulation of actin cytoskeleton | 0.17152591 | 1.00E+00 | 5.74E-01 | 2.34E-01 |
| LightCyan | Starch and sucrose metabolism | 0.175233792 | 1.00E+00 | 5.74E-01 | 2.34E-01 |
| LightCyan | Glutathione metabolism | 0.175233792 | 1.00E+00 | 5.74E-01 | 2.34E-01 |
| LightCyan | Glycerophospholipid metabolism | 0.294780763 | 1.00E+00 | 5.74E-01 | 3.63E-01 |
| LightCyan | Calcium signaling pathway | 0.361261619 | 1.00E+00 | 5.74E-01 | 4.13E-01 |
| LightCyan | Tight junction | 0.398361945 | 1.00E+00 | 5.74E-01 | 4.25E-01 |
| LightCyan | MAPK signaling pathway | 0.574068366 | 1.00E+00 | 5.74E-01 | 5.74E-01 |
| LightYellow | MAPK signaling pathway | 0.005359369 | 1.07E-02 | 1.07E-02 | 1.07E-02 |
| LightYellow | Regulation of actin cytoskeleton | 0.133064277 | 2.66E-01 | 1.33E-01 | 1.33E-01 |
| MidnightBlue | Nitrobenzene degradation | 0.000712208 | 3.13E-02 | 3.13E-02 | 3.13E-02 |
| MidnightBlue | Androgen and estrogen metabolism | 0.004136645 | 1.82E-01 | 1.78E-01 | 9.10E-02 |
| MidnightBlue | Histidine metabolism | 0.010011526 | 4.41E-01 | 4.20E-01 | 1.21E-01 |
| MidnightBlue | Tryptophan metabolism | 0.012705517 | 5.59E-01 | 5.21E-01 | 1.21E-01 |
| MidnightBlue | Carbon fixation | 0.01379091 | 6.07E-01 | 5.52E-01 | 1.21E-01 |
| MidnightBlue | Notch signaling pathway | 0.0228888 | 1.00E+00 | 7.88E-01 | 1.21E-01 |
| MidnightBlue | Tyrosine metabolism | 0.0228888 | 1.00E+00 | 7.88E-01 | 1.21E-01 |
| MidnightBlue | Stilbene, coumarine and lignin biosynthesis | 0.027538726 | 1.00E+00 | 7.88E-01 | 1.21E-01 |
| MidnightBlue | 1,4-Dichlorobenzene degradation | 0.027538726 | 1.00E+00 | 7.88E-01 | 1.21E-01 |
| MidnightBlue | Styrene degradation | 0.027538726 | 1.00E+00 | 7.88E-01 | 1.21E-01 |
| MidnightBlue | Methane metabolism | 0.054365244 | 1.00E+00 | 7.88E-01 | 2.17E-01 |
| MidnightBlue | Porphyrin and chlorophyll metabolism | 0.080496775 | 1.00E+00 | 7.88E-01 | 2.36E-01 |
| MidnightBlue | Aminophosphonate metabolism | 0.080496775 | 1.00E+00 | 7.88E-01 | 2.36E-01 |
| MidnightBlue | 1- and 2-Methylnaphthalene degradation | 0.080496775 | 1.00E+00 | 7.88E-01 | 2.36E-01 |
| MidnightBlue | Bisphenol A degradation | 0.080496775 | 1.00E+00 | 7.88E-01 | 2.36E-01 |
| MidnightBlue | Bile acid biosynthesis | 0.105950151 | 1.00E+00 | 7.88E-01 | 2.59E-01 |
| MidnightBlue | Glyoxylate and dicarboxylate metabolism | 0.105950151 | 1.00E+00 | 7.88E-01 | 2.59E-01 |
| MidnightBlue | Pentose phosphate pathway | 0.105950151 | 1.00E+00 | 7.88E-01 | 2.59E-01 |
| MidnightBlue | Limonene and pinene degradation | 0.130741828 | 1.00E+00 | 7.88E-01 | 2.73E-01 |
| MidnightBlue | Cysteine metabolism | 0.130741828 | 1.00E+00 | 7.88E-01 | 2.73E-01 |
| MidnightBlue | Phenylalanine metabolism | 0.154887888 | 1.00E+00 | 7.88E-01 | 2.73E-01 |
| MidnightBlue | Dorso-ventral axis formation | 0.154887888 | 1.00E+00 | 7.88E-01 | 2.73E-01 |
| MidnightBlue | N-Glycan biosynthesis | 0.154887888 | 1.00E+00 | 7.88E-01 | 2.73E-01 |
| MidnightBlue | Glycine, serine and threonine metabolism | 0.154887888 | 1.00E+00 | 7.88E-01 | 2.73E-01 |
| MidnightBlue | Alanine and aspartate metabolism | 0.154887888 | 1.00E+00 | 7.88E-01 | 2.73E-01 |
| MidnightBlue | Selenoamino acid metabolism | 0.178404051 | 1.00E+00 | 7.88E-01 | 2.77E-01 |
| MidnightBlue | Glutamate metabolism | 0.178404051 | 1.00E+00 | 7.88E-01 | 2.77E-01 |
| MidnightBlue | Propanoate metabolism | 0.178404051 | 1.00E+00 | 7.88E-01 | 2.77E-01 |
| MidnightBlue | gamma-Hexachlorocyclohexane degradation | 0.201305681 | 1.00E+00 | 7.88E-01 | 2.77E-01 |
| MidnightBlue | Alzheimer's disease | 0.201305681 | 1.00E+00 | 7.88E-01 | 2.77E-01 |
| MidnightBlue | Biosynthesis of steroids | 0.201305681 | 1.00E+00 | 7.88E-01 | 2.77E-01 |
| MidnightBlue | Biosynthesis of Steroids | 0.201305681 | 1.00E+00 | 7.88E-01 | 2.77E-01 |
| MidnightBlue | Lysine degradation | 0.287062079 | 1.00E+00 | 7.88E-01 | 3.71E-01 |
| MidnightBlue | Pyruvate metabolism | 0.287062079 | 1.00E+00 | 7.88E-01 | 3.71E-01 |
| MidnightBlue | Regulation of actin cytoskeleton | 0.302901892 | 1.00E+00 | 7.88E-01 | 3.81E-01 |
| MidnightBlue | TGF-beta signaling pathway | 0.326627583 | 1.00E+00 | 7.88E-01 | 3.98E-01 |
| MidnightBlue | MAPK signaling pathway | 0.334671632 | 1.00E+00 | 7.88E-01 | 3.98E-01 |
| MidnightBlue | Adherens junction | 0.364127387 | 1.00E+00 | 7.88E-01 | 4.22E-01 |
| MidnightBlue | Glycolysis / Gluconeogenesis | 0.416723602 | 1.00E+00 | 7.88E-01 | 4.58E-01 |
| MidnightBlue | Gap junction | 0.416723602 | 1.00E+00 | 7.88E-01 | 4.58E-01 |
| MidnightBlue | Insulin signaling pathway | 0.524019247 | 1.00E+00 | 7.88E-01 | 5.49E-01 |
| MidnightBlue | Tight junction | 0.524019247 | 1.00E+00 | 7.88E-01 | 5.49E-01 |
| MidnightBlue | Focal adhesion | 0.737587931 | 1.00E+00 | 7.88E-01 | 7.55E-01 |
| MidnightBlue | Cytokine-cytokine receptor interaction | 0.788091226 | 1.00E+00 | 7.88E-01 | 7.88E-01 |
| Purple | Insulin signaling pathway | 0.041520011 | 1.00E+00 | 8.26E-01 | 3.82E-01 |
| Purple | Neuroactive ligand-receptor interaction | 0.041520011 | 1.00E+00 | 8.26E-01 | 3.82E-01 |
| Purple | Phosphatidylinositol signaling system | 0.04980987 | 1.00E+00 | 8.26E-01 | 3.82E-01 |
| Purple | Pantothenate and CoA biosynthesis | 0.061054068 | 1.00E+00 | 8.26E-01 | 3.82E-01 |
| Purple | Prion disease | 0.090244097 | 1.00E+00 | 8.26E-01 | 3.82E-01 |
| Purple | Aminophosphonate metabolism | 0.090244097 | 1.00E+00 | 8.26E-01 | 3.82E-01 |
| Purple | Wnt signaling pathway | 0.093649758 | 1.00E+00 | 8.26E-01 | 3.82E-01 |
| Purple | Gap junction | 0.113531896 | 1.00E+00 | 8.26E-01 | 3.82E-01 |
| Purple | Streptomycin biosynthesis | 0.118575596 | 1.00E+00 | 8.26E-01 | 3.82E-01 |
| Purple | Calcium signaling pathway | 0.156202232 | 1.00E+00 | 8.26E-01 | 4.53E-01 |
| Purple | Hedgehog signaling pathway | 0.198653893 | 1.00E+00 | 8.26E-01 | 4.64E-01 |
| Purple | Jak-STAT signaling pathway | 0.201609186 | 1.00E+00 | 8.26E-01 | 4.64E-01 |
| Purple | Notch signaling pathway | 0.248166987 | 1.00E+00 | 8.26E-01 | 4.64E-01 |
| Purple | Aminosugars metabolism | 0.248166987 | 1.00E+00 | 8.26E-01 | 4.64E-01 |
| Purple | Starch and sucrose metabolism | 0.271826068 | 1.00E+00 | 8.26E-01 | 4.64E-01 |
| Purple | ABC transporters - Organism-specific | 0.271826068 | 1.00E+00 | 8.26E-01 | 4.64E-01 |
| Purple | Apoptosis | 0.271826068 | 1.00E+00 | 8.26E-01 | 4.64E-01 |
| Purple | Galactose metabolism | 0.294780763 | 1.00E+00 | 8.26E-01 | 4.75E-01 |
| Purple | Fructose and mannose metabolism | 0.317050844 | 1.00E+00 | 8.26E-01 | 4.84E-01 |
| Purple | Ribosome | 0.379943386 | 1.00E+00 | 8.26E-01 | 5.50E-01 |
| Purple | Toll-like receptor signaling pathway | 0.399662501 | 1.00E+00 | 8.26E-01 | 5.50E-01 |
| Purple | Focal adhesion | 0.424734047 | 1.00E+00 | 8.26E-01 | 5.50E-01 |
| Purple | Glycerophospholipid metabolism | 0.437337607 | 1.00E+00 | 8.26E-01 | 5.50E-01 |
| Purple | Glycolysis / Gluconeogenesis | 0.455326813 | 1.00E+00 | 8.26E-01 | 5.50E-01 |
| Purple | Purine metabolism | 0.552363598 | 1.00E+00 | 8.26E-01 | 6.32E-01 |
| Purple | Tight junction | 0.566855424 | 1.00E+00 | 8.26E-01 | 6.32E-01 |
| Purple | Regulation of actin cytoskeleton | 0.728516065 | 1.00E+00 | 8.26E-01 | 7.82E-01 |
| Purple | MAPK signaling pathway | 0.754769626 | 1.00E+00 | 8.26E-01 | 7.82E-01 |
| Purple | Cytokine-cytokine receptor interaction | 0.826014824 | 1.00E+00 | 8.26E-01 | 8.26E-01 |
| Red | Ribosome | 0.000305408 | 2.87E-02 | 2.87E-02 | 2.87E-02 |
| Red | Proteasome | 0.002897702 | 2.72E-01 | 2.69E-01 | 1.36E-01 |
| Red | Prion disease | 0.011787296 | 1.00E+00 | 9.99E-01 | 2.59E-01 |
| Red | RNA polymerase | 0.011787296 | 1.00E+00 | 9.99E-01 | 2.59E-01 |
| Red | Purine metabolism | 0.013755708 | 1.00E+00 | 9.99E-01 | 2.59E-01 |
| Red | Huntington's disease | 0.026858882 | 1.00E+00 | 9.99E-01 | 4.21E-01 |
| Red | Pyrimidine metabolism | 0.034589808 | 1.00E+00 | 9.99E-01 | 4.64E-01 |
| Red | Alzheimer's disease | 0.085259789 | 1.00E+00 | 9.99E-01 | 9.91E-01 |
| Red | Lysine degradation | 0.114982959 | 1.00E+00 | 9.99E-01 | 9.91E-01 |
| Red | Folate biosynthesis | 0.132719551 | 1.00E+00 | 9.99E-01 | 9.91E-01 |
| Red | Reductive carboxylate cycle (CO2 fixation) | 0.132719551 | 1.00E+00 | 9.99E-01 | 9.91E-01 |
| Red | N-Glycan biosynthesis | 0.136465918 | 1.00E+00 | 9.99E-01 | 9.91E-01 |
| Red | Starch and sucrose metabolism | 0.175364299 | 1.00E+00 | 9.99E-01 | 9.91E-01 |
| Red | Selenoamino acid metabolism | 0.200085654 | 1.00E+00 | 9.99E-01 | 9.91E-01 |
| Red | Citrate cycle (TCA cycle) | 0.226243284 | 1.00E+00 | 9.99E-01 | 9.91E-01 |
| Red | Neurodegenerative Disorders | 0.226243284 | 1.00E+00 | 9.99E-01 | 9.91E-01 |
| Red | Ganglioside biosynthesis | 0.226243284 | 1.00E+00 | 9.99E-01 | 9.91E-01 |
| Red | Retinol metabolism | 0.228915663 | 1.00E+00 | 9.99E-01 | 9.91E-01 |
| Red | Biotin metabolism | 0.228915663 | 1.00E+00 | 9.99E-01 | 9.91E-01 |
| Red | Keratan sulfate biosynthesis | 0.228915663 | 1.00E+00 | 9.99E-01 | 9.91E-01 |
| Red | Circadian rhythm | 0.228915663 | 1.00E+00 | 9.99E-01 | 9.91E-01 |
| Red | Arginine and proline metabolism | 0.268981173 | 1.00E+00 | 9.99E-01 | 9.91E-01 |
| Red | Glycerolipid metabolism | 0.285875539 | 1.00E+00 | 9.99E-01 | 9.91E-01 |
| Red | Adherens junction | 0.293656787 | 1.00E+00 | 9.99E-01 | 9.91E-01 |
| Red | Globoside metabolism | 0.322630541 | 1.00E+00 | 9.99E-01 | 9.91E-01 |
| Red | Aminosugars metabolism | 0.340000871 | 1.00E+00 | 9.99E-01 | 9.91E-01 |
| Red | Notch signaling pathway | 0.340000871 | 1.00E+00 | 9.99E-01 | 9.91E-01 |
| Red | Glycerophospholipid metabolism | 0.396490656 | 1.00E+00 | 9.99E-01 | 9.91E-01 |
| Red | N-Glycan degradation | 0.405733278 | 1.00E+00 | 9.99E-01 | 9.91E-01 |
| Red | Sulfur metabolism | 0.405733278 | 1.00E+00 | 9.99E-01 | 9.91E-01 |
| Red | Aminoacyl-tRNA biosynthesis | 0.405733278 | 1.00E+00 | 9.99E-01 | 9.91E-01 |
| Red | Pantothenate and CoA biosynthesis | 0.405733278 | 1.00E+00 | 9.99E-01 | 9.91E-01 |
| Red | beta-Alanine metabolism | 0.405733278 | 1.00E+00 | 9.99E-01 | 9.91E-01 |
| Red | Glycosaminoglycan degradation | 0.405733278 | 1.00E+00 | 9.99E-01 | 9.91E-01 |
| Red | Glycine, serine and threonine metabolism | 0.415712852 | 1.00E+00 | 9.99E-01 | 9.91E-01 |
| Red | Urea cycle and metabolism of amino groups | 0.415712852 | 1.00E+00 | 9.99E-01 | 9.91E-01 |
| Red | Alanine and aspartate metabolism | 0.415712852 | 1.00E+00 | 9.99E-01 | 9.91E-01 |
| Red | Dentatorubropallidoluysian atrophy (DRPLA) | 0.415712852 | 1.00E+00 | 9.99E-01 | 9.91E-01 |
| Red | Nitrogen metabolism | 0.415712852 | 1.00E+00 | 9.99E-01 | 9.91E-01 |
| Red | Galactose metabolism | 0.478677423 | 1.00E+00 | 9.99E-01 | 9.91E-01 |
| Red | O-Glycan biosynthesis | 0.501963731 | 1.00E+00 | 9.99E-01 | 9.91E-01 |
| Red | Chondroitin sulfate biosynthesis | 0.501963731 | 1.00E+00 | 9.99E-01 | 9.91E-01 |
| Red | Glutamate metabolism | 0.501963731 | 1.00E+00 | 9.99E-01 | 9.91E-01 |
| Red | Pentose and glucuronate interconversions | 0.542240142 | 1.00E+00 | 9.99E-01 | 9.91E-01 |
| Red | Phenylalanine, tyrosine and tryptophan biosynthesis | 0.542240142 | 1.00E+00 | 9.99E-01 | 9.91E-01 |
| Red | Basal transcription factors | 0.542240142 | 1.00E+00 | 9.99E-01 | 9.91E-01 |
| Red | Bisphenol A degradation | 0.542240142 | 1.00E+00 | 9.99E-01 | 9.91E-01 |
| Red | Porphyrin and chlorophyll metabolism | 0.542240142 | 1.00E+00 | 9.99E-01 | 9.91E-01 |
| Red | High-mannose type N-glycan biosynthesis | 0.542240142 | 1.00E+00 | 9.99E-01 | 9.91E-01 |
| Red | DNA polymerase | 0.542240142 | 1.00E+00 | 9.99E-01 | 9.91E-01 |
| Red | Insulin signaling pathway | 0.569949761 | 1.00E+00 | 9.99E-01 | 9.91E-01 |
| Red | Wnt signaling pathway | 0.570814104 | 1.00E+00 | 9.99E-01 | 9.91E-01 |
| Red | gamma-Hexachlorocyclohexane degradation | 0.579624583 | 1.00E+00 | 9.99E-01 | 9.91E-01 |
| Red | Glycosphingolipid metabolism | 0.579624583 | 1.00E+00 | 9.99E-01 | 9.91E-01 |
| Red | Valine, leucine and isoleucine degradation | 0.579624583 | 1.00E+00 | 9.99E-01 | 9.91E-01 |
| Red | Glyoxylate and dicarboxylate metabolism | 0.647572427 | 1.00E+00 | 9.99E-01 | 9.99E-01 |
| Red | Methionine metabolism | 0.647572427 | 1.00E+00 | 9.99E-01 | 9.99E-01 |
| Red | Blood group glycolipid biosynthesis - neo-lactoseries | 0.647572427 | 1.00E+00 | 9.99E-01 | 9.99E-01 |
| Red | Blood group glycolipid biosynthesis - lactoseries | 0.647572427 | 1.00E+00 | 9.99E-01 | 9.99E-01 |
| Red | Pentose phosphate pathway | 0.647572427 | 1.00E+00 | 9.99E-01 | 9.99E-01 |
| Red | Gap junction | 0.666694213 | 1.00E+00 | 9.99E-01 | 9.99E-01 |
| Red | Apoptosis | 0.707470776 | 1.00E+00 | 9.99E-01 | 9.99E-01 |
| Red | Nicotinate and nicotinamide metabolism | 0.707470776 | 1.00E+00 | 9.99E-01 | 9.99E-01 |
| Red | ABC transporters - Organism-specific | 0.707470776 | 1.00E+00 | 9.99E-01 | 9.99E-01 |
| Red | Glutathione metabolism | 0.707470776 | 1.00E+00 | 9.99E-01 | 9.99E-01 |
| Red | Glycosylphosphatidylinositol(GPI)-anchor biosynthesis | 0.728807899 | 1.00E+00 | 9.99E-01 | 9.99E-01 |
| Red | Cysteine metabolism | 0.728807899 | 1.00E+00 | 9.99E-01 | 9.99E-01 |
| Red | Nucleotide sugars metabolism | 0.728807899 | 1.00E+00 | 9.99E-01 | 9.99E-01 |
| Red | Tight junction | 0.748762724 | 1.00E+00 | 9.99E-01 | 9.99E-01 |
| Red | Focal adhesion | 0.767013541 | 1.00E+00 | 9.99E-01 | 9.99E-01 |
| Red | Phenylalanine metabolism | 0.791426908 | 1.00E+00 | 9.99E-01 | 9.99E-01 |
| Red | Dorso-ventral axis formation | 0.791426908 | 1.00E+00 | 9.99E-01 | 9.99E-01 |
| Red | Hedgehog signaling pathway | 0.839670771 | 1.00E+00 | 9.99E-01 | 9.99E-01 |
| Red | Propanoate metabolism | 0.839670771 | 1.00E+00 | 9.99E-01 | 9.99E-01 |
| Red | Carbon fixation | 0.839670771 | 1.00E+00 | 9.99E-01 | 9.99E-01 |
| Red | Glycolysis / Gluconeogenesis | 0.848927835 | 1.00E+00 | 9.99E-01 | 9.99E-01 |
| Red | TGF-beta signaling pathway | 0.867706833 | 1.00E+00 | 9.99E-01 | 9.99E-01 |
| Red | Oxidative phosphorylation | 0.867706833 | 1.00E+00 | 9.99E-01 | 9.99E-01 |
| Red | Tyrosine metabolism | 0.905411692 | 1.00E+00 | 9.99E-01 | 9.99E-01 |
| Red | Fatty acid metabolism | 0.905411692 | 1.00E+00 | 9.99E-01 | 9.99E-01 |
| Red | Calcium signaling pathway | 0.927776801 | 1.00E+00 | 9.99E-01 | 9.99E-01 |
| Red | Regulation of actin cytoskeleton | 0.928905129 | 1.00E+00 | 9.99E-01 | 9.99E-01 |
| Red | Tryptophan metabolism | 0.954978475 | 1.00E+00 | 9.99E-01 | 9.99E-01 |
| Red | ECM-receptor interaction | 0.954978475 | 1.00E+00 | 9.99E-01 | 9.99E-01 |
| Red | MAPK signaling pathway | 0.955827582 | 1.00E+00 | 9.99E-01 | 9.99E-01 |
| Red | Phosphatidylinositol signaling system | 0.957307604 | 1.00E+00 | 9.99E-01 | 9.99E-01 |
| Red | Fructose and mannose metabolism | 0.957307604 | 1.00E+00 | 9.99E-01 | 9.99E-01 |
| Red | Neuroactive ligand-receptor interaction | 0.959944474 | 1.00E+00 | 9.99E-01 | 9.99E-01 |
| Red | Cell cycle | 0.959944474 | 1.00E+00 | 9.99E-01 | 9.99E-01 |
| Red | Hematopoietic cell lineage | 0.959944474 | 1.00E+00 | 9.99E-01 | 9.99E-01 |
| Red | Jak-STAT signaling pathway | 0.967282662 | 1.00E+00 | 9.99E-01 | 9.99E-01 |
| Red | Toll-like receptor signaling pathway | 0.985329537 | 1.00E+00 | 9.99E-01 | 9.99E-01 |
| Red | Complement and coagulation cascades | 0.997092772 | 1.00E+00 | 9.99E-01 | 9.99E-01 |
| Red | Cytokine-cytokine receptor interaction | 0.999496445 | 1.00E+00 | 9.99E-01 | 9.99E-01 |
| Salmon | ATP synthesis | 0.042438823 | 4.24E-01 | 3.20E-01 | 1.73E-01 |
| Salmon | Glutamate metabolism | 0.059005325 | 5.90E-01 | 3.20E-01 | 1.73E-01 |
| Salmon | Cytokine-cytokine receptor interaction | 0.068291093 | 6.83E-01 | 3.20E-01 | 1.73E-01 |
| Salmon | Butanoate metabolism | 0.07534174 | 7.53E-01 | 3.20E-01 | 1.73E-01 |
| Salmon | TGF-beta signaling pathway | 0.115191888 | 1.00E+00 | 3.20E-01 | 1.73E-01 |
| Salmon | Oxidative phosphorylation | 0.115191888 | 1.00E+00 | 3.20E-01 | 1.73E-01 |
| Salmon | Adherens junction | 0.130741828 | 1.00E+00 | 3.20E-01 | 1.73E-01 |
| Salmon | Wnt signaling pathway | 0.138434378 | 1.00E+00 | 3.20E-01 | 1.73E-01 |
| Salmon | Jak-STAT signaling pathway | 0.212406867 | 1.00E+00 | 3.20E-01 | 2.36E-01 |
| Salmon | MAPK signaling pathway | 0.32012347 | 1.00E+00 | 3.20E-01 | 3.20E-01 |

**Figure 1.** KEGG diagrams of the pathways described in the text. The p-values for the corresponding pathways can be found in Table 1. The enzymatic reactions that are found in mice are highlighted by coloring the boxes green. The white boxes are not found in mice. The boxes with red outlines and red text are genes found in the module. By following the red boxes through the pathways one may gain insights regarding the module function. Boxes in green that are not highlighted in red outlines may be present in the mouse liver but may not be present in the list of filtered genes.

Blue Module

### Brown Module

### Green Module

### GreenYellow Module

### LightYellow Module

### MidnightBlue Module

### Red Module

**Table 2.** This table maps Locus Link IDs to their respective KEGG Pathway ID.

| **Locus Link ID** | **KEGG Pathway ID** |
| --- | --- |
| 16773 | mmu04510; mmu04512; |
| 16772 | mmu04510; mmu04512; mmu05060; |
| 16775 | mmu04510; mmu04512; |
| 12928 | mmu04010; mmu04510; mmu04810; mmu04910; |
| 74185 | mmu00500; |
| 13198 | mmu04010; |
| 83428 | mmu04080; |
| 12350 | mmu00910; |
| 19699 | mmu04510; mmu04512; |
| 12359 | mmu00380; mmu00680; mmu05030; |
| 12827 | mmu04510; mmu04512; |
| 12826 | mmu04510; mmu04512; |
| 20742 | mmu04530; |
| 14387 | mmu00052; mmu00500; |
| 214579 | mmu00251; mmu00650; |
| 21991 | mmu00010; mmu00031; mmu00051; mmu00710; |
| 11637 | mmu00230; |
| 72590 | mmu00623; mmu00650; mmu00960; |
| 12502 | mmu04640; |
| 12500 | mmu04640; |
| 56050 | mmu00100; mmu00120; mmu00150; mmu00340; mmu00350; mmu00360; mmu00361; mmu00363; mmu00380; mmu00624; mmu00626; mmu00627; mmu00643; mmu00680; mmu00903; mmu00940; mmu80100; |
| 12504 | mmu04640; |
| 12505 | mmu04512; mmu04640; |
| 17773 | mmu04080; |
| 12509 | mmu04610; mmu04640; |
| 70737 | mmu04530; |
| 18777 | mmu00564; |
| 14257 | mmu04060; |
| 19354 | mmu04010; mmu04310; mmu04510; mmu04520; mmu04620; mmu04810; |
| 18778 | mmu00564; mmu00590; mmu04010; |
| 16177 | mmu04010; mmu04060; mmu04210; mmu04640; |
| 16176 | mmu04010; mmu04060; mmu04210; mmu04620; mmu04640; |
| 19211 | mmu00562; mmu04070; mmu04510; mmu04530; |
| 11937 | mmu04020; |
| 67041 | mmu00072; mmu00280; mmu00650; |
| 218203 | mmu00380; |
| 60363 | mmu04530; |
| 19193 | mmu00260; mmu00310; |
| 14425 | mmu00512; |
| 14815 | mmu04080; |
| 108100 | mmu04520; mmu04810; mmu05050; |
| 16197 | mmu04060; mmu04630; mmu04640; |
| 56458 | mmu04910; |
| 56752 | mmu00310; |
| 20116 | mmu03010; |
| 14537 | mmu00512; |
| 56452 | mmu04110; |
| 53973 | mmu00071; mmu00361; mmu00380; |
| 56454 | mmu00220; |
| 24064 | mmu04630; |
| 16452 | mmu04630; |
| 13026 | mmu00440; mmu00564; |
| 211347 | mmu00770; |
| 14042 | mmu00532; |
| 22340 | mmu04060; mmu04510; |
| 110460 | mmu00062; mmu00071; mmu00072; mmu00280; mmu00310; mmu00380; mmu00620; mmu00632; mmu00640; mmu00650; |
| 15926 | mmu00020; mmu00480; mmu00720; |
| 94041 | mmu00230; |
| 81535 | mmu00600; |
| 12649 | mmu04110; |
| 18416 | mmu00220; mmu00330; |
| 18414 | mmu04060; mmu04630; |
| 68603 | mmu00100; mmu80100; |
| 15109 | mmu00340; mmu00910; |
| 66447 | mmu00480; |
| 108072 | mmu04080; mmu04540; |
| 22095 | mmu04080; |
| 319229 | mmu04080; |
| 13099 | mmu00071; mmu00361; mmu00380; |
| 105148 | mmu00290; mmu00970; |
| 13096 | mmu00071; mmu00361; mmu00380; |
| 13094 | mmu00071; mmu00361; mmu00380; |
| 319554 | mmu00100; mmu00900; mmu80100; |
| 269941 | mmu00532; |
| 12865 | mmu00190; |
| 12867 | mmu00190; |
| 12866 | mmu00190; |
| 16832 | mmu00010; mmu00272; mmu00620; mmu00640; |
| 12862 | mmu00190; |
| 27405 | mmu02011; |
| 57319 | mmu00530; |
| 20848 | mmu04630; |
| 20849 | mmu04630; |
| 20846 | mmu04620; mmu04630; |
| 12156 | mmu04060; mmu04340; mmu04350; |
| 26357 | mmu02011; |
| 17305 | mmu04330; |
| 26358 | mmu00830; |
| 218397 | mmu04010; mmu05040; |
| 11949 | mmu00190; mmu00193; |
| 26408 | mmu04010; |
| 12388 | mmu04520; |
| 66935 | mmu04330; |
| 12260 | mmu04610; |
| 68262 | mmu00561; mmu00564; |
| 60322 | mmu00532; |
| 114641 | mmu03010; |
| 20019 | mmu00230; mmu00240; mmu03020; |
| 70789 | mmu00380; |
| 24063 | mmu04630; |
| 223628 | mmu00140; mmu00150; |
| 142980 | mmu04620; |
| 67089 | mmu03050; |
| 14661 | mmu00220; mmu00251; mmu00330; mmu00910; |
| 16548 | mmu00051; |
| 21949 | mmu04060; |
| 15368 | mmu00860; |
| 16542 | mmu04060; mmu04510; |
| 66354 | mmu04330; |
| 12160 | mmu04340; mmu04350; |
| 56386 | mmu00051; mmu00510; mmu00512; mmu00513; mmu00561; mmu00563; mmu00600; mmu00601; mmu00602; mmu00603; mmu00604; |
| 12164 | mmu04340; mmu04350; |
| 78405 | mmu04010; |
| 67130 | mmu00190; |
| 71586 | mmu00500; mmu00790; |
| 14186 | mmu04010; mmu04810; |
| 14187 | mmu00040; mmu00051; mmu00052; mmu00561; mmu00620; |
| 13806 | mmu00010; mmu00400; |
| 170442 | mmu00310; |
| 54405 | mmu00190; |
| 14347 | mmu00051; mmu00510; mmu00512; mmu00513; mmu00561; mmu00563; mmu00600; mmu00601; mmu00602; mmu00603; mmu00604; |
| 15212 | mmu00511; mmu00530; mmu00531; mmu00603; |
| 30878 | mmu04080; |
| 11636 | mmu00230; |
| 18193 | mmu00310; |
| 14867 | mmu00480; |
| 21832 | mmu04640; |
| 14863 | mmu00480; |
| 14862 | mmu00480; |
| 78928 | mmu00563; |
| 110606 | mmu00510; |
| 27409 | mmu02011; |
| 21936 | mmu04060; |
| 12766 | mmu04060; |
| 57266 | mmu04060; |
| 57914 | mmu04060; mmu04630; |
| 27219 | mmu00562; mmu00632; mmu00760; mmu04070; |
| 12442 | mmu04020; mmu04110; |
| 99586 | mmu00240; mmu00410; mmu00770; |
| 22235 | mmu00040; mmu00500; mmu00520; |
| 22234 | mmu00600; |
| 66204 | mmu00010; mmu00620; mmu00632; |
| 18128 | mmu04320; mmu04330; |
| 14718 | mmu00251; mmu00252; mmu00272; mmu00330; mmu00350; mmu00360; mmu00400; mmu00401; mmu00710; mmu00950; |
| 67374 | mmu04530; |
| 18679 | mmu04910; |
| 107029 | mmu00620; |
| 12908 | mmu00252; |
| 12613 | mmu00120; mmu00561; |
| 20336 | mmu04530; |
| 67305 | mmu00480; |
| 20620 | mmu00562; mmu00632; mmu00760; mmu04070; |
| 56360 | mmu00632; mmu00903; |
| 17260 | mmu04010; |
| 83964 | mmu04530; |
| 93960 | mmu04310; |
| 66979 | mmu00230; mmu00240; mmu03030; |
| 16765 | mmu04010; |
| 107995 | mmu04110; |
| 26446 | mmu03050; |
| 26441 | mmu03050; |
| 15211 | mmu00511; mmu00530; mmu00531; mmu00603; |
| 19116 | mmu04060; mmu04080; mmu04630; |
| 54140 | mmu04020; mmu04080; |
| 54635 | mmu04060; mmu04510; mmu04540; |
| 110135 | mmu04610; |
| 12831 | mmu04510; mmu04512; |
| 17901 | mmu04810; |
| 75320 | mmu00564; |
| 20750 | mmu04510; mmu04512; |
| 107476 | mmu00061; mmu00620; mmu00640; mmu04910; |
| 74246 | mmu00052; mmu00520; |
| 18643 | mmu04810; |
| 170483 | mmu04080; |
| 12534 | mmu04020; mmu04110; mmu04540; |
| 13849 | mmu00625; |
| 18645 | mmu04810; |
| 12530 | mmu04110; |
| 26921 | mmu04010; |
| 11474 | mmu04510; mmu04520; mmu04530; mmu04810; |
| 16987 | mmu00100; mmu80100; |
| 56217 | mmu04530; |
| 23939 | mmu04010; mmu04540; |
| 16334 | mmu04810; mmu04910; mmu05050; |
| 16164 | mmu04060; mmu04630; |
| 19166 | mmu03050; |
| 67054 | mmu00230; |
| 14825 | mmu04060; |
| 17246 | mmu00380; mmu04110; |
| 21877 | mmu00240; |
| 21871 | mmu00190; mmu00193; |
| 14129 | mmu04640; |
| 243537 | mmu00340; |
| 16180 | mmu04060; mmu04210; |
| 108079 | mmu04910; |
| 16185 | mmu04060; mmu04630; |
| 22271 | mmu00240; |
| 63953 | mmu04010; |
| 19064 | mmu04080; |
| 56421 | mmu00010; mmu00030; mmu00051; mmu00052; mmu04910; |
| 66355 | mmu00230; |
| 15483 | mmu00140; mmu00150; |
| 18791 | mmu04610; |
| 53867 | mmu04510; mmu04512; |
| 72349 | mmu04010; |
| 70266 | mmu00380; |
| 14756 | mmu00563; |
| 14751 | mmu00010; mmu00030; mmu00500; |
| 23871 | mmu04320; |
| 76441 | mmu04310; |
| 12892 | mmu00860; |
| 23971 | mmu00230; mmu00450; mmu00920; |
| 21809 | mmu04010; mmu04060; mmu04110; mmu04350; |
| 67512 | mmu00561; mmu00564; |
| 16333 | mmu04810; mmu04910; mmu05050; |
| 19944 | mmu03010; |
| 268449 | mmu03010; |
| 11472 | mmu04510; mmu04520; mmu04530; mmu04810; |
| 94192 | mmu00512; |
| 57257 | mmu04510; mmu04810; |
| 268697 | mmu04020; mmu04110; |
| 56637 | mmu04110; mmu04310; mmu04340; mmu04510; mmu04910; mmu05010; |
| 381903 | mmu00510; |
| 68671 | mmu00440; mmu00564; |
| 22612 | mmu04520; mmu04530; |
| 14430 | mmu00052; mmu00520; |
| 18018 | mmu04020; mmu04310; |
| 14433 | mmu00010; mmu01510; mmu05010; mmu05040; mmu05050; |
| 71562 | mmu00380; mmu00630; |
| 19017 | mmu04910; |
| 18947 | mmu00561; |
| 20091 | mmu03010; |
| 20090 | mmu03010; |
| 319625 | mmu00010; |
| 27214 | mmu04110; |
| 12877 | mmu04320; |
| 108682 | mmu00251; mmu00252; mmu00710; |
| 20303 | mmu04060; mmu04620; |
| 20302 | mmu04060; mmu04620; |
| 20305 | mmu04060; |
| 20304 | mmu04060; mmu04620; |
| 20306 | mmu04060; |
| 17129 | mmu04350; |
| 20652 | mmu00120; |
| 21788 | mmu04610; |
| 12978 | mmu04060; mmu04640; |
| 17311 | mmu04060; mmu04640; |
| 12444 | mmu04110; mmu04310; mmu04510; mmu04630; |
| 12577 | mmu04110; |
| 12576 | mmu04110; |
| 16411 | mmu04810; |
| 17319 | mmu00350; mmu00360; |
| 17252 | mmu00051; mmu00052; mmu00053; mmu00120; mmu00260; mmu00310; mmu00363; mmu00520; mmu00564; mmu00625; mmu00632; mmu00650; |
| 214951 | mmu04320; |
| 110196 | mmu00100; mmu00900; mmu80100; |
| 237310 | mmu04060; mmu04630; |
| 18720 | mmu00562; mmu04070; mmu04810; |
| 319448 | mmu04512; |
| 54485 | mmu04330; |
| 16192 | mmu04060; mmu04630; mmu04640; |
| 19241 | mmu04810; |
| 67092 | mmu00220; mmu00260; mmu00330; |
| 15452 | mmu00230; |
| 15114 | mmu05040; |
| 11307 | mmu02011; |
| 17380 | mmu04640; mmu05010; |
| 16476 | mmu04010; mmu04310; mmu04510; mmu04620; |
| 14677 | mmu04530; mmu04540; |
| 14674 | mmu04810; |
| 14678 | mmu04530; mmu04540; |
| 15275 | mmu00010; mmu00051; mmu00052; mmu00500; mmu00521; mmu00530; mmu04910; |
| 16478 | mmu04010; |
| 11656 | mmu00260; |
| 18590 | mmu04010; mmu04060; mmu04510; mmu04540; mmu04810; |
| 66480 | mmu03010; |
| 18596 | mmu04010; mmu04020; mmu04060; mmu04510; mmu04540; mmu04810; |
| 18595 | mmu04010; mmu04020; mmu04060; mmu04510; mmu04540; mmu04810; |
| 27050 | mmu03010; |
| 66489 | mmu03010; |
| 18751 | mmu04010; mmu04020; mmu04070; mmu04310; mmu04510; mmu04530; mmu04540; |
| 16590 | mmu04060; mmu04640; |
| 54393 | mmu04080; |
| 18205 | mmu04010; |
| 102093 | mmu04910; |
| 16971 | mmu05010; |
| 110208 | mmu00030; |
| 99730 | mmu03022; |
| 26874 | mmu02011; |
| 268930 | mmu04110; |
| 11950 | mmu00190; mmu00193; |
| 16154 | mmu04060; mmu04630; |
| 109821 | mmu04610; |
| 50798 | mmu00530; |
| 11720 | mmu00271; mmu00450; |
| 329165 | mmu04810; |
| 21926 | mmu04010; mmu04060; mmu04210; mmu04350; mmu04620; mmu04640; |
| 15978 | mmu04060; mmu04350; mmu04630; |
| 21925 | mmu04020; |
| 13909 | mmu00960; |
| 27226 | mmu00564; |
| 59031 | mmu00532; mmu00920; |
| 13618 | mmu04020; mmu04080; |
| 104112 | mmu00020; |
| 14081 | mmu00071; |
| 20133 | mmu00230; mmu00240; |
| 18113 | mmu00760; |
| 53897 | mmu00600; |
| 13435 | mmu00271; |
| 20299 | mmu04060; |
| 13120 | mmu00071; mmu00361; mmu00380; |
| 20296 | mmu04060; |
| 233733 | mmu00512; |
| 14884 | mmu03022; |
| 12628 | mmu04610; |
| 12390 | mmu04510; |
| 241226 | mmu04510; mmu04512; mmu04810; |
| 12622 | mmu04310; |
| 18578 | mmu00230; |
| 18472 | mmu00564; |
| 17000 | mmu04060; |
| 67993 | mmu00760; |
| 74147 | mmu00062; mmu00071; mmu00280; mmu00310; mmu00380; mmu00410; mmu00632; mmu00640; mmu00650; mmu00903; mmu00930; |
| 116939 | mmu00280; mmu00310; mmu00340; mmu00350; mmu00360; mmu00561; mmu00564; mmu00624; mmu00632; mmu00642; mmu00650; mmu00903; mmu00960; |
| 20229 | mmu00330; |
| 18577 | mmu00230; |
| 17215 | mmu04110; |
| 17217 | mmu04110; |
| 17216 | mmu04110; |
| 17219 | mmu04110; |
| 12481 | mmu04640; |
| 71934 | mmu00910; |
| 18973 | mmu00230; mmu00240; mmu03030; |
| 110147 | mmu00310; |
| 15159 | mmu00860; |
| 20044 | mmu03010; |
| 12974 | mmu00020; mmu00630; |
| 12802 | mmu04080; |
| 66211 | mmu03010; |
| 110382 | mmu04610; |
| 17174 | mmu04610; |
| 22165 | mmu04510; |
| 17850 | mmu00280; mmu00640; |
| 12483 | mmu04640; |
| 11611 | mmu00252; mmu00260; |
| 11964 | mmu00190; mmu00193; |
| 103988 | mmu00010; mmu00051; mmu00052; mmu00500; mmu00521; mmu00530; mmu04910; |
| 107747 | mmu00670; |
| 12524 | mmu04620; |
| 18655 | mmu00010; mmu00710; |
| 58988 | mmu04350; mmu04910; |
| 58861 | mmu04020; mmu04080; |
| 12292 | mmu04020; |
| 20963 | mmu04020; |
| 23945 | mmu00561; |
| 216558 | mmu00040; mmu00052; mmu00500; mmu00520; |
| 18806 | mmu00564; |
| 286940 | mmu04010; mmu04510; |
| 21881 | mmu00030; mmu00710; |
| 14526 | mmu04080; |
| 16408 | mmu04810; |
| 269378 | mmu00271; mmu00450; |
| 50880 | mmu00450; |
| 14933 | mmu00561; |
| 20541 | mmu04020; |
| 384783 | mmu04910; |
| 11761 | mmu00280; mmu00350; mmu00380; mmu00750; mmu00760; |
| 192195 | mmu04530; |
| 22262 | mmu00230; |
| 13822 | mmu04530; |
| 13823 | mmu04530; |
| 17886 | mmu04530; mmu04810; |
| 18787 | mmu04610; |
| 54342 | mmu00251; mmu00530; |
| 18783 | mmu00564; mmu00590; mmu04010; |
| 114716 | mmu04630; |
| 14630 | mmu00251; mmu00480; |
| 19650 | mmu04110; mmu04350; |
| 13360 | mmu00100; mmu80100; |
| 16790 | mmu00480; mmu04640; |
| 14068 | mmu04610; |
| 14067 | mmu04610; |
| 14062 | mmu04020; mmu04080; mmu04610; mmu04810; |
| 11461 | mmu04510; mmu04520; mmu04530; mmu04810; |
| 11465 | mmu04510; mmu04520; mmu04530; mmu04810; |
| 14598 | mmu00430; mmu00450; mmu00460; mmu00480; mmu00590; |
| 21813 | mmu04010; mmu04060; mmu04350; mmu04520; |
| 14595 | mmu00051; mmu00052; mmu00510; mmu00512; mmu00513; mmu00533; mmu00561; mmu00563; mmu00600; mmu00601; mmu00602; mmu00603; mmu00604; |
| 14594 | mmu00601; mmu00602; |
| 14590 | mmu00790; |
| 12702 | mmu04630; mmu04910; |
| 12703 | mmu04630; mmu04910; |
| 233571 | mmu04080; |
| 12709 | mmu00220; mmu00330; |
| 192289 | mmu00310; |
| 66136 | mmu00230; mmu00240; mmu03020; |
| 56720 | mmu00380; |
| 18024 | mmu05060; |
| 208846 | mmu04310; |
| 14447 | mmu00010; |
| 11867 | mmu04810; |
| 18538 | mmu04110; |
| 12995 | mmu04310; mmu04520; mmu04530; |
| 22137 | mmu00562; mmu00632; mmu00760; mmu04070; |
| 57342 | mmu04510; |
| 24099 | mmu04060; |
| 234356 | mmu00532; |
| 14858 | mmu00480; |
| 12843 | mmu04510; mmu04512; |
| 12842 | mmu04510; mmu04512; |
| 16816 | mmu00564; |
| 12847 | mmu04080; |
| 60525 | mmu00010; mmu00620; mmu00640; mmu00720; |
| 20311 | mmu04060; |
| 18441 | mmu04080; |
| 15565 | mmu04020; mmu04080; |
| 16780 | mmu04510; mmu04512; |
| 12362 | mmu01510; mmu04010; mmu05040; mmu05050; |
| 12363 | mmu04010; |
| 12367 | mmu01510; mmu04010; mmu04210; mmu05010; mmu05040; mmu05050; |
| 12364 | mmu04210; |
| 105855 | mmu04810; |
| 20928 | mmu02011; |
| 114663 | mmu00521; mmu00562; mmu04070; |
| 226414 | mmu00252; mmu00970; |
| 235339 | mmu00010; mmu00620; |
| 15163 | mmu04530; |
| 15162 | mmu04510; |
| 225192 | mmu04080; |
| 15510 | mmu05060; |
| 20322 | mmu00051; |
| 11303 | mmu02011; |
| 75572 | mmu00010; mmu00620; mmu00632; |
| 11722 | mmu00500; |
| 14645 | mmu00251; mmu00550; mmu00910; |
| 53357 | mmu00564; mmu00590; mmu04010; |
| 19299 | mmu02011; |
| 19294 | mmu04520; |
| 192156 | mmu00100; mmu80100; |
| 17768 | mmu00630; mmu00670; |
| 56040 | mmu03010; |
| 226548 | mmu04330; mmu05010; |
| 66142 | mmu00190; |
| 107869 | mmu00260; mmu00271; mmu00272; mmu00450; mmu00910; |
| 13867 | mmu04020; |
| 18746 | mmu00010; mmu00230; mmu00620; mmu00710; mmu04910; |
| 66495 | mmu00190; |
| 22420 | mmu04310; mmu04340; |
| 19224 | mmu00590; |
| 76408 | mmu02011; |
| 11429 | mmu00020; mmu00630; mmu00720; |
| 50787 | mmu00532; |
| 15945 | mmu04060; mmu04620; |
| 99470 | mmu04530; |
| 14969 | mmu04640; |
| 12741 | mmu04530; |
| 12425 | mmu04020; mmu04080; |
| 59027 | mmu00760; |
| 26394 | mmu00564; |
| 208449 | mmu00600; |
| 18103 | mmu00230; mmu00240; |
| 98878 | mmu00240; mmu00670; |
| 12428 | mmu04110; |
| 19046 | mmu04510; mmu04810; mmu04910; |
| 19982 | mmu03010; |
| 76709 | mmu04810; |
| 15469 | mmu00150; mmu00340; mmu00350; mmu00380; mmu00440; mmu00450; mmu00626; |
| 19766 | mmu04210; |
| 19988 | mmu03010; |
| 14563 | mmu04060; mmu04350; |
| 23959 | mmu00230; mmu00240; mmu00760; |
| 12986 | mmu04060; mmu04630; mmu04640; |
| 13035 | mmu04080; |
| 22370 | mmu04510; mmu04512; |
| 22371 | mmu04510; mmu04512; mmu04610; |
| 59029 | mmu03050; |
| 234724 | mmu00350; mmu00360; mmu00400; mmu00401; mmu00950; |
| 78600 | mmu00230; |
| 74637 | mmu00562; mmu00632; mmu00760; mmu04070; |
| 22084 | mmu04910; |
| 140557 | mmu04110; |
| 56613 | mmu04010; |
| 66102 | mmu04060; |
| 22772 | mmu04340; |
| 50785 | mmu00532; |
| 66454 | mmu00760; |
| 109905 | mmu04010; mmu04510; |
| 208624 | mmu00510; |
| 17194 | mmu04610; |
| 15277 | mmu00010; mmu00051; mmu00052; mmu00500; mmu00521; mmu00530; mmu04910; |
| 68202 | mmu00190; |
| 54598 | mmu04080; |
| 60596 | mmu00230; mmu04540; |
| 18968 | mmu00230; mmu00240; mmu03030; |
| 16440 | mmu04020; mmu04070; mmu04540; |
| 16828 | mmu00010; mmu00272; mmu00620; mmu00640; |
| 53627 | mmu04310; |
| 338375 | mmu00190; mmu00193; |
| 20775 | mmu00100; mmu00900; mmu80100; |
| 13136 | mmu04610; mmu04640; |
| 20852 | mmu04630; |
| 12495 | mmu00230; mmu00240; |
| 12490 | mmu04640; |
| 12491 | mmu04512; mmu04640; |
| 67005 | mmu00230; mmu00240; mmu03020; |
| 12499 | mmu00230; mmu00240; |
| 18708 | mmu04070; mmu04210; mmu04510; mmu04620; mmu04630; mmu04810; mmu04910; |
| 241656 | mmu04510; mmu04810; |
| 20971 | mmu04512; |
| 18817 | mmu04110; |
| 26413 | mmu04010; mmu04320; mmu04350; mmu04510; mmu04520; mmu04540; mmu04810; mmu04910; |
| 26410 | mmu04010; |
| 22153 | mmu04540; |
| 19263 | mmu04520; |
| 21416 | mmu04310; mmu04520; |
| 15139 | mmu04610; |
| 66190 | mmu00600; |
| 20700 | mmu04610; |
| 110391 | mmu00790; |
| 16414 | mmu04810; |
| 20703 | mmu04610; |
| 21894 | mmu04510; |
| 15980 | mmu04060; mmu04630; |
| 21898 | mmu04620; |
| 56175 | mmu05010; |
| 69019 | mmu00310; mmu00780; |
| 27375 | mmu04530; |
| 56012 | mmu00010; |
| 20411 | mmu04520; mmu04910; |
| 27279 | mmu04060; |
| 27373 | mmu04310; mmu04340; mmu04540; mmu04710; |
| 17436 | mmu00620; mmu00710; |
| 56150 | mmu04110; |
| 18140 | mmu00380; |
| 14198 | mmu00230; |
| 18223 | mmu04330; |
| 16956 | mmu00561; mmu05010; |
| 14609 | mmu04540; |
| 83602 | mmu03022; |
| 14179 | mmu04010; mmu04810; |
| 224105 | mmu04010; mmu04510; mmu04810; |
| 18750 | mmu04010; mmu04020; mmu04070; mmu04310; mmu04510; mmu04530; mmu04540; |
| 76884 | mmu04810; |
| 56508 | mmu04010; |
| 11554 | mmu04020; mmu04080; mmu04540; |
| 11416 | mmu00604; |
| 17897 | mmu04810; |
| 192663 | mmu02011; |
| 21824 | mmu04610; |
| 21823 | mmu00350; mmu05020; |
| 12774 | mmu04060; |
| 12773 | mmu04060; |
| 12475 | mmu04010; mmu04620; mmu04640; mmu04810; |
| 56717 | mmu04910; |
| 11811 | mmu00791; |
| 18132 | mmu04320; mmu04330; |
| 14109 | mmu03010; |
| 14104 | mmu00061; mmu00062; mmu04910; |
| 11898 | mmu00220; mmu00252; mmu00330; |
| 330260 | mmu00361; mmu00363; |
| 14103 | mmu04010; mmu04060; mmu04210; |
| 13101 | mmu00071; mmu00361; mmu00380; |
| 240672 | mmu04010; |
| 12982 | mmu04060; mmu04630; mmu04640; |
| 22142 | mmu04540; |
| 57296 | mmu03050; |
| 16155 | mmu04060; mmu04630; |
| 12633 | mmu04210; |
| 24088 | mmu04620; |
| 72039 | mmu00280; |
| 74596 | mmu00564; mmu04070; |
| 78894 | mmu00650; |
| 170743 | mmu04620; |
| 72136 | mmu00272; |
| 12914 | mmu01510; mmu04020; mmu04110; mmu04310; mmu04330; mmu04350; mmu04520; mmu04630; mmu05040; |
| 109754 | mmu00530; |
| 56378 | mmu04810; |
| 13171 | mmu00280; mmu00310; mmu00340; mmu00350; mmu00360; mmu00564; mmu00624; mmu00632; mmu00642; mmu00650; mmu00903; mmu00960; |
| 56373 | mmu04610; |
| 18451 | mmu00330; |
| 13179 | mmu04350; |
| 12262 | mmu04610; |

THE END
