# Supplementary material for: Integrating Genetic and Network Analysis to Characterize Genes Related to Mouse Weight
Source: PLoS Genet. 2006 Aug 18;2(8):e130. doi: 10.1371/journal.pgen.0020130 (PMC1550283; doi:10.1371/journal.pgen.0020130)
Supplement: Supplementary file 7 [file pgen.0020130.st001.doc]

Supplementary Table S1. Characterisitics of the female liver gene co-expression modules.

| **Module** | **Gene Content** | **Mean k_in_** | **Significant GO Catagories** |
| --- | --- | --- | --- |
| all | 3421 | 13.53 | N/A |
| black | 548 | 17.1 | Nucleus |
| blue | 534 | 8.26 | Extracellular Space, Extracellular Matrix, Basement Membrane |
| brown | 366 | 23.29 | Mitochondrion |
| cyan | 96 | 10.82 | Cytoskeleton |
| green | 406 | 12.73 | Plasma Membrane, Lysosome |
| greenyellow | 121 | 10.15 | Nucleus (chromosomal Maintenance) |
| light cyan | 119 | 13.3 | Extracellular Space |
| light yellow | 35 | 9.16 | None |
| midnight blue | 84 | 6.6 | Intracellular |
| purple | 139 | 4.24 | None |
| red | 772 | 16.67 | Intracellular Membrane Bound Organelle |
| salmon | 98 | 6.17 | None |
