# Supplementary material for: Integrating Genetic and Network Analysis to Characterize Genes Related to Mouse Weight
Source: PLoS Genet. 2006 Aug 18;2(8):e130. doi: 10.1371/journal.pgen.0020130 (PMC1550283; doi:10.1371/journal.pgen.0020130)
Supplement: Supplementary file 8 [file pgen.0020130.st002.doc]

Supplementary Table S2. Summary of Blue Module hub genes and their properties

| **Gene Symbol** | **Genbank Accession** | **T-test p-value^1^** | **Connectivity^2^** |
| --- | --- | --- | --- |
| Fscn1 | NM_007984 | 0.003 | 14.9 |
| Rcn3 | ri\|0610034O06\|R000004I22\|\|1380 | 0.003 | 18.4 |
| Arhgap18 | AK014728 | 0.006 | 16.1 |
| Pdgfrb | NM_008809 | 0.010 | 14.2 |
| 2310016C16Rik | AK009378 | 0.010 | 22.6 |
| 4632428N05Rik | NM_028732 | 0.013 | 16.7 |
| Col6a3 | AF064749 | 0.015 | 18.8 |
| Col14a1 | AK009906 | 0.018 | 18.2 |
| C1qr1 | NM_010740 | 0.018 | 19.6 |
| Rgs3 | NM_019492 | 0.018 | 12.8 |
| Col16a1 | AK012212 | 0.024 | 13.0 |
| Nid1 | NM_010917 | 0.024 | 15.0 |
| Pcolce | NM_008788 | 0.026 | 17.5 |
| 2610001E17Rik | AK011256 | 0.027 | 22.8 |
| Anxa2 | AK012563 | 0.031 | 26.5 |
| Plk2 | M96163 | 0.034 | 14.7 |
| Tubb4 | AK013717 | 0.035 | 12.0 |
| Atp8b2 | AF156546 | 0.036 | 24.5 |
| Lama2 | U12147 | 0.042 | 16.7 |
| Col4a1 | J04694 | 0.043 | 11.9 |
| Mest | NM_008590 | 0.045 | 23.3 |
| Col1a1 | U08020 | 0.046 | 17.8 |
| Heph | NM_010417 | 0.047 | 14.8 |
| Loxl1 | BC003973 | 0.048 | 11.7 |
| Rgs10 | NM_026418 | 0.050 | 11.7 |
| Adamts2 | AW476152 | 0.052 | 12.1 |
| Itih3 | NM_008407 | 0.056 | 20.7 |
| Fhl2 | NM_010212 | 0.057 | 14.3 |
| 2810489O06Rik | ri\|2810489O06\|ZX00067F14\|\|1560 | 0.057 | 16.2 |
| Gpr105 | AF177211 | 0.059 | 13.3 |
| 1200015A22Rik | AK004778 | 0.060 | 12.4 |
| 1200009O22Rik | AK004681 | 0.060 | 12.3 |
| Col1a2 | NM_007743 | 0.062 | 13.6 |
| Pdgfrb | BB437264 | 0.065 | 14.2 |
| Fbln2 | NM_007992 | 0.067 | 15.1 |
| Slc43a1 | AK011417 | 0.068 | 14.3 |
| Anxa5 | NM_009673 | 0.069 | 24.1 |
| Fbn1 | L29454 | 0.071 | 18.6 |
| 3222401M22Rik | AK014318 | 0.080 | 14.1 |
| Lrat | NM_023624 | 0.082 | 13.1 |
| Agpt2 | AK019860 | 0.084 | 12.5 |
| Igfbp7 | AK002277 | 0.090 | 19.7 |
| Serpinf2 | NM_008878 | 0.093 | 15.5 |
| Adamts2 | BB208731 | 0.098 | 12.1 |
| **Gene Symbol** | **Genbank Accession** | **t-test P-value^1^** | **Connectivity^2^** |
| 2310046G15Rik | AK009847 | 0.102 | 17.4 |
| Col1a2 | BB138762 | 0.102 | 13.6 |
| Gpld1 | NM_008156 | 0.103 | 14.3 |
| Col4a2 | J04695 | 0.105 | 16.0 |
| 1110039C07Rik | AK004171 | 0.110 | 12.9 |
| Tulp3 | NM_011657 | 0.114 | 15.9 |
| Thbs2 | NM_011581 | 0.115 | 20.9 |
| Serpina1d | NM_009246 | 0.123 | 13.4 |
| Sparc | AK014286 | 0.123 | 18.3 |
| Fetub | AK009310 | 0.124 | 16.6 |
| Ddah2 | NM_016765 | 0.128 | 23.2 |
| 1500041B16Rik | AK019934 | 0.128 | 16.9 |
| Akr1b8 | NM_008012 | 0.130 | 14.7 |
| Map4k4 | NM_008696 | 0.133 | 14.4 |
| Gpm6b | AF254875 | 0.135 | 12.6 |
| Eml1 | AK003593 | 0.138 | 20.1 |
| Fabp4 | BC002148 | 0.140 | 12.8 |
| Lxn | NM_016753 | 0.143 | 19.4 |
| Matn2 | NM_016762 | 0.143 | 14.4 |
| E130307J04Rik | AI452306 | 0.144 | 12.2 |
| 1500041B16Rik | AK014216 | 0.204 | 16.9 |
| Rgs3 | AK004648 | 0.211 | 12.8 |
| Pam | NM_013626 | 0.215 | 13.0 |
| Rasip1 | AK011557 | 0.220 | 15.3 |
| Tfpi | AF016313 | 0.225 | 12.9 |
| 5730528L13Rik | AK013199 | 0.225 | 16.3 |
| 5031439A09Rik | AK019886 | 0.227 | 21.1 |
| Ppic | NM_008908 | 0.237 | 35.5 |
| Fgb | AK011118 | 0.250 | 15.7 |
| Map4k4 | BB162626 | 0.256 | 14.4 |
| Rcn3 | AK021299 | 0.270 | 18.4 |
| Pdir | AK012415 | 0.283 | 18.2 |
| Mat1a | L13622 | 0.283 | 13.1 |
| Tulp3 | AK009482 | 0.286 | 15.9 |
| Sgce | NM_011360 | 0.298 | 12.1 |
| Dll4 | NM_019454 | 0.312 | 18.0 |
| Armcx1 | AK019405 | 0.313 | 13.1 |
| Chst7 | NM_021715 | 0.330 | 20.7 |
| Gmpr | AK002326 | 0.332 | 13.4 |
| 0 | J03953 | 0.336 | 12.7 |
| F7 | NM_010172 | 0.343 | 26.4 |
| Trfr2 | AK004965 | 0.345 | 15.4 |
| 1300018K11Rik | AK005049 | 0.371 | 16.0 |
| 9430028I06Rik | ri\|9430028I06\|PX00108K24\|\|1318 | 0.372 | 16.0 |
| Icam2 | NM_010494 | 0.382 | 13.9 |
| **Gene Symbol** | **Genbank Accession** | **t-test P-value^1^** | **Connectivity^2^** |
| Sh3d4 | NM_011366 | 0.392 | 13.9 |
| Sprr1a | NM_009264 | 0.413 | 12.8 |
| Serpina3n | NM_009252 | 0.451 | 14.0 |
| Proz | NM_025834 | 0.458 | 14.7 |
| Ang1 | NM_007447 | 0.494 | 16.0 |
| Ramp2 | NM_019444 | 0.503 | 16.1 |
| C8b | AK015340 | 0.510 | 16.6 |
| Armcx2 | NM_026139 | 0.511 | 22.0 |
| Tm4sf6 | ri\|6720473L21\|PX00060G18\|\|1635 | 0.513 | 16.2 |
| C1qr1 | BB075734 | 0.530 | 19.6 |
| Maged2 | NM_030700 | 0.550 | 17.0 |
| Lama2 | BB221368 | 0.554 | 16.7 |
| C1qr1 | AV295373 | 0.554 | 19.6 |
| Col14a1 | AK017553 | 0.569 | 18.2 |
| Serpina3m | NM_009253 | 0.583 | 12.5 |
| 5730485H21Rik | AK017709 | 0.583 | 16.8 |
| 2600017H02Rik | AK011240 | 0.586 | 13.2 |
| AI324046 | D78343 | 0.589 | 21.5 |
| AI173486 | AI850690 | 0.593 | 19.4 |
| Msx2 | NM_013601 | 0.605 | 17.8 |
| Rnase4 | NM_021472 | 0.608 | 13.1 |
| Esam1 | AK009223 | 0.608 | 17.1 |
| Gne | NM_015828 | 0.609 | 11.7 |
| Tulp3 | BE954025 | 0.629 | 15.9 |
| Emcn | NM_016885 | 0.687 | 23.0 |
| Nrarp | AK012426 | 0.693 | 18.9 |
| F2r | NM_010169 | 0.726 | 12.7 |
| Tek | NM_013690 | 0.767 | 11.9 |
| Fsp27 | M61737 | 0.799 | 14.5 |
| Gpihbp1 | AK003305 | 0.806 | 13.7 |
| Atp8b2 | BE986744 | 0.812 | 24.5 |
| 1200003C23Rik | AV248982 | 0.836 | 14.4 |
| 1200003C23Rik | NM_025809 | 0.862 | 14.4 |
| 1110039C07Rik | AI481202 | 0.884 | 12.9 |
| Epb4.1l2 | AJ245854 | 0.893 | 22.9 |
| Itih1 | NM_008406 | 0.935 | 18.3 |
| Fbln2 | AK017870 | 0.960 | 15.1 |
| Bucs1 | AW319694 | 0.963 | 11.7 |

^1^P-value for standard T-test for corresponding gene expression between top and bottom quartile mice according to weight in independent C57BL/6J x DBA/2J F2 cross consisting of 111 animals

^2^Connectivity refers to within module connectivity (k^in^)
