# Supplementary material for: Integrating Genetic and Network Analysis to Characterize Genes Related to Mouse Weight
Source: PLoS Genet. 2006 Aug 18;2(8):e130. doi: 10.1371/journal.pgen.0020130 (PMC1550283; doi:10.1371/journal.pgen.0020130)
Supplement: Supplementary file 9 [file pgen.0020130.st003.doc]

Supplementary Table S3. Regressing body weight on multiple mQTLs (SNPs) of the blue module.

| **Genetic Model** | **%variance explained** | **Model p-value (F)** |
| --- | --- | --- |
| **Weight~Chr1+Chr5+Chr15+Chr19+Chr1*Chr5+Chr1*Chr15+**  **Chr1*Chr19+Chr5*Chr15+Chr5*Chr19+Chr15*Chr19** | **53.5** | **7.01 e-07** |
| **Weight~Chr1+Chr5+Chr15+Chr19+Chr1*Chr15+Chr1*Chr19** | **44.9** | **3.69 e-09** |
| **Weight~Chr1+Chr5+Chr15+Chr19** | **34.0** | **1.46 e-08** |
